# Acidosis-mediated increase in IFN-γ-induced PD-L1 expression on cancer cells as an immune escape mechanism in solid tumors

**DOI:** 10.1186/s12943-023-01900-0

**Published:** 2023-12-15

**Authors:** Philipp Knopf, Dimitri Stowbur, Sabrina H. L. Hoffmann, Natalie Hermann, Andreas Maurer, Valentina Bucher, Marilena Poxleitner, Bredi Tako, Dominik Sonanini, Balaji Krishnamachary, Sanhita Sinharay, Birgit Fehrenbacher, Irene Gonzalez-Menendez, Felix Reckmann, David Bomze, Lukas Flatz, Daniela Kramer, Martin Schaller, Stephan Forchhammer, Zaver M. Bhujwalla, Leticia Quintanilla-Martinez, Klaus Schulze-Osthoff, Mark D. Pagel, Marieke F. Fransen, Martin Röcken, André F. Martins, Bernd J. Pichler, Kamran Ghoreschi, Manfred Kneilling

**Affiliations:** 1 Werner Siemens Imaging Center, Department of Preclinical Imaging and Radiopharmacy, Eberhard Karls University, Tübingen, Germany; 2 Present Address: Cluster of Excellence iFIT (EXC 2180) “Image Guided and Functionally Instructed Tumor Therapies”, Röntgenweg 13, 72076 Tübingen, Germany; 3 Division of Cancer Imaging Research, The Russell H Morgan Department of Radiology and Radiological Science, The Johns Hopkins University School of Medicine, Baltimore, MD USA; 4 Department of Cancer Systems Imaging, MD Anderson Cancer Center, 1881 East Rd, Houston, TX 77054 USA; 5 Department of Dermatology, Eberhard Karls University, Tübingen, Germany; 6 Institute of Pathology and Neuropathology, Department of Pathology, Eberhard Karls University of Tübingen and Comprehensive Cancer Center, Tübingen University Hospital, Tübingen, Germany; 7 Department of Dermatology, Tel-Aviv Medical Center, Tel-Aviv, Israel; 8 Interfaculty Institute of Biochemistry, Eberhard Karls University, Tübingen, Germany; 9 Sidney Kimmel Comprehensive Cancer Center, The Johns Hopkins University, School of Medicine, Baltimore, MD USA; 10 Department of Radiation Oncology and Molecular Radiation Sciences, The Johns Hopkins University, School of Medicine, Baltimore, MD USA; 11 German Cancer Consortium (DKTK), partner site Tübingen, German Cancer Research Center (DKFZ), Im Neuenheimer Feld 280, 69120 Heidelberg, Germany; 12 Department of Immunohematology and Blood Transfusion, Leiden University Medical Center (LUMC), Leiden, Netherlands; 13 Department of Dermatology, Venereology and Allergology, Charité – Universitätsmedizin Berlin, Corporate Member of Freie Universität Berlin, Humboldt-Universität zu Berlin, Berlin Institute of Health, 10117 Berlin, Germany

**Keywords:** Modulators of tumor microenvironment, Immune checkpoint inhibitors, Drug resistance mechanisms in immunotherapy, pH modulation of the tumor microenvironment, Immunotherapy, Oncology, T-cells, Checkpoint inhibitors, Immune response, Biomarkers, Combination therapy, Precision medicine

## Abstract

Immune checkpoint inhibitors have revolutionized cancer therapy, yet the efficacy of these treatments is often limited by the heterogeneous and hypoxic tumor microenvironment (TME) of solid tumors. In the TME, programmed death-ligand 1 (PD-L1) expression on cancer cells is mainly regulated by Interferon-gamma (IFN-γ), which induces T cell exhaustion and enables tumor immune evasion. In this study, we demonstrate that acidosis, a common characteristic of solid tumors, significantly increases IFN-γ-induced PD-L1 expression on aggressive cancer cells, thus promoting immune escape. Using preclinical models, we found that acidosis enhances the genomic expression and phosphorylation of signal transducer and activator of transcription 1 (STAT1), and the translation of STAT1 mRNA by eukaryotic initiation factor 4F (elF4F), resulting in an increased PD-L1 expression. We observed this effect in murine and human anti-PD-L1-responsive tumor cell lines, but not in anti-PD-L1-nonresponsive tumor cell lines. In vivo studies fully validated our in vitro findings and revealed that neutralizing the acidic extracellular tumor pH by sodium bicarbonate treatment suppresses IFN-γ-induced PD-L1 expression and promotes immune cell infiltration in responsive tumors and thus reduces tumor growth. However, this effect was not observed in anti-PD-L1-nonresponsive tumors. In vivo experiments in tumor-bearing IFN-γ^−/−^ mice validated the dependency on immune cell-derived IFN-γ for acidosis-mediated cancer cell PD-L1 induction and tumor immune escape. Thus, acidosis and IFN-γ-induced elevation of PD-L1 expression on cancer cells represent a previously unknown immune escape mechanism that may serve as a novel biomarker for anti-PD-L1/PD-1 treatment response. These findings have important implications for the development of new strategies to enhance the efficacy of immunotherapy in cancer patients.

**Supplementary Information:**

The online version contains supplementary material available at 10.1186/s12943-023-01900-0.

## Background

Treatment with the programmed cell death-protein 1 (PD-1) immune checkpoint-targeting monoclonal antibodies (mAbs) pembrolizumab or nivolumab has significantly improved the overall survival of patients with various types of cancer. PD-1/PD-L1 blockade represents an approved standard-of-care treatment for patients with metastatic melanoma or non-small cell lung cancer [1, 2]. Most recently, further PD-1 blocking mAbs have been approved for treatment of patients with advanced squamous cell skin cancer and basal cell carcinomas as well as for patients with advanced or recurrent DNA mismatch repair deficient (dMMR) endometrial cancers [3–5].

Despite the impressive improvement in anti-PD-1 therapy for advanced melanoma, only 16% of patients achieve a complete response, and 25% achieve a partial response. Anti-PD-1 treatment results in an estimated 5-year overall survival of 34% in all patients [1], highlighting the importance of studying the mechanisms of treatment response and resistance.

Reliable and robust biomarkers to predict the response to checkpoint inhibitor therapy remain elusive. Key elements that influence the success of checkpoint inhibitor therapies are immune cell infiltration and the tumor mutational burden [6, 7]. A nonrandomized phase Ib clinical trial with pembrolizumab identified an activated T cell gene expression profile and PD-1 expression as a favorable prognostic biomarker [7]. Several studies suggest that patients with tumors overexpressing PD-L1 exhibit a better clinical outcome to anti-PD-L1 therapy [8]. PD-L1 expression on cancer cells can vary between different cancer types. Cell-intrinsic, genetic characteristics of the respective cancer cells, such as 9p24.1 amplification leading to increased Janus kinase 2 (JAK2) and PD-L1 expression [9] or oncogenic RAS stabilizing the *Pdl1* mRNA [10], explain differences in PD-L1 expression among tumor types. Nevertheless, tumors lacking PD-L1 expression on cancer cells still respond to anti-PD-L1 therapy, indicating that the expression of PD-L1 on cancer cells and tumor-infiltrating immune cells might prevent an antitumor CD8^+^ T cell response [11–13]. In addition to cancer cell-intrinsic, genetic PD-L1 regulation, cancer cell-extrinsic factors regulate PD-L1 expression. These factors include IFN-γ secreted by tumor-infiltrating T lymphocytes [14] or hypoxia [15] within the tumor microenvironment (TME). Therefore, cancer cell-intrinsic PD-L1 expression alone might be a poor biomarker to predict the response to PD-1/PD-L1 blockade. Dynamic changes in PD-L1 expression patterns in the TME and infiltrating immune cells may be more relevant for immune escape and predicting the therapy response [13, 16].

Resistance to treatment caused by tumor heterogeneity, clonal cooperation [17], and immune inhibition [18] by elements such as acidosis within the TME [19] represent a major challenge in checkpoint inhibitor therapies. Various immune cells, such as T cells, natural killer cells, dendritic cells, and macrophages, show impaired effector functions in an acidic TME, representing a mechanism of tumor immune escape and checkpoint inhibitor resistance [20]. Furthermore, lactate produced by cancer cells has been reported to increase PD-L1 expression in human lung cancer cells [21]. Knockdown of lactate dehydrogenase-A by a small hairpin RNA increased the efficacy of anti-PD-1 therapy by inducing CD4^+^ or CD8^+^ T cell and NK cell recruitment to the tumor [22]. A recent study by Kwon et al*.* suggested that acidosis increases PD-L1 expression via STAT3 activation in MDA-MB-231 cells [23]. Further, tumor acidosis suppresses IFN-γ secretion by immune cells representing a separate mechanism of tumor immune escape [24]. Hypoxia is often associated with the rapid growth of solid tumors and, triggers the upregulation of hypoxia-inducible factor-1α (HIF-1α). This in turn leads to increased PD-L1 expression in tumor cells. Concurrently, hypoxia-induced acidosis activates multiple signaling pathways, further promoting PD-L1 expression and dampening antitumor immune responses [25–27]. Necrotic regions within the tumor microenvironment release proinflammatory mediators such as TNF and damage-associated molecular patterns (DAMPs), which in turn stimulate immune cells to upregulate PD-L1 to safeguard against excessive T cell activation [28–30]. The intricate connection between hypoxia, acidosis, necrosis, and PD-L1 expression fosters immune escape by hindering effector T cell function and promoting the expansion of regulatory T cells [31, 32].

The TME is heterogeneous in terms of spatial and temporal dynamic immune cell infiltration and distribution [17]. IFN-γ secretion by immune cells, including T cells, B cells, NK cells, and antigen-presenting cells, induces PD-L1 expression by binding to the interferon type II receptor, leading to JAK1, JAK2 and STAT1 activation by phosphorylation [14]. IFN-γ-induced PD-L1 expression on the cell membrane is mediated by transcriptional induction and subsequent translation of the *Stat1* mRNA by the eukaryotic translation initiation complex eIF4F. Consequently, we hypothesize that immune cell-derived IFN-γ within a tumor region with a neutral tumor pH_e_ affects the dynamics of PD-L1 expression in adjacent acidic tumor regions and thus might represent a novel immune escape mechanism.

To our knowledge, nothing is known so far concerning the effect of IFN-γ in combination with tumor acidosis on PD-L1 expression as an additional prognostic biomarker. In the present study, we show that acidosis increased IFN-γ-induced PD-L1 expression in cancer cells both in vitro and in vivo. Acidosis-induced PD-L1 expression was mediated by increased STAT1 activity, which relied on the efficient translation of the *Stat1* mRNA by elF4F. Furthermore, neutralization of the acidic tumor pH_e_ not only suppressed PD-L1 expression but also increased immune cell recruitment and focal tumor necrosis. Our results highlight a critical role for tumor acidosis in the IFN-γ-mediated induction of PD-L1 expression via enhanced STAT1 phosphorylation and subsequent immune escape of anti-PD-L1 responsive tumors.

## Methods

### Cell lines and reagents

The MC38^wt^, MC38^PD−L1−/−^ (generated by CRISPR/Cas9 [12]) and human MCF-7, MIA-PaCa-2, SK-MEL-28 and U87 MG cell lines were maintained in DMEM (Biochrom, Berlin, Germany) with 3.7 g l^−1^ NaHCO_3_ supplemented with 10% fetal bovine serum (FBS, Sigma-Aldrich, St. Louis, MO, USA), 100 U ml^−1^ penicillin–streptomycin (Biochrom) and 10 mM 4-(2-hydroxyethyl)-1-piperazineethanesulfonic acid (HEPES, Biochrom). HCA-7 colony 29 cells (Werner Siemens Imaging Center (WSIC) originally from ATCC) were maintained in DMEM with 3.7 g l^−1^ NaHCO_3_ supplemented with 10% FBS, 100 U ml^−1^ penicillin–streptomycin and 1 mM sodium pyruvate (Biochrom). B16-F10^wt^ (ATCC), MCF-7 (WSIC originally from ATCC) and U87 MG (kindly provided by Simone Fulda) cell lines were maintained in DMEM with 3.7 g l^−1^ NaHCO_3_ supplemented with 10% FBS and 100 U ml^−1^ penicillin–streptomycin. CT26^wt^ and CT26^PD−L1−/−^ (generated by CRISPR/Cas9 [12]) cell lines, as well as the 4T1^wt^ cell line (WSIC originally from ATCC), were maintained in RPMI 1640 (Biochrom) with 2.0 g l^−1^ NaHCO_3_ supplemented with 10% FBS and 100 U ml^−1^ penicillin–streptomycin. For pH_e_-defined cell culture media, DMEM (Biochrom) with 3.7 g l^−1^ (44.05 mM, neutral) or 0.34 g l^−1^ (4 mM, acidic) NaHCO_3_ and RPMI 1640 (Sigma-Aldrich) with 3.7 g l^−1^ (44.05 mM, neutral) or 0.08 g l^−1^ (1 mM, acidic) NaHCO_3_ were used. Cells were maintained at 37 °C with 5% CO_2_ in a humidified incubator and tested for mycoplasma monthly.

Murine IFN-γ was purchased from Merck Millipore (Billerica, MA, USA), and human IFN-γ was purchased from R&D Systems (Minneapolis, MN, USA). Lipofectamine 2000 and Opti-MEM were obtained from Thermo Fisher Scientific (Waltham, MA, USA). Silvestrol was purchased from MedChemExpress (Monmouth Junction, NJ, USA), and phosphatase inhibitor cocktails 2 (P5726) and 3 (P0044) were purchased from Sigma-Aldrich. Protease inhibitor cocktail (04693159001) was purchased from Roche (Basel, Switzerland). Antibodies against STAT1 (9172), pSTAT1 (phospho Y701; 9167), KA-PD-L1 (13684), PD-L1 (13684), elF4A1 (2490), elF4E (2067) and β-actin (4970) were purchased from Cell Signaling Technology (Danvers, MA, USA). The anti-PD-L1 (AF1019) antibody was purchased from R&D Systems, anti-pSTAT1 (phospho Y701; 29045) was purchased from Abcam (Cambridge, UK), and anti-Ki67 (14–5698) was purchased from eBioscience (San Diego, CA, USA).

### In vitro cell culture

For murine MC38^wt^ and B16-F10^wt^ cells unbuffered DMEM media was supplemented with 10% FBS, 100 U ml^−1^ penicillin–streptomycin and NaHCO_3_. An acidic pH_e_ of 6.8 for the continuative in vitro experiments with murine MC38^wt^ and B16-F10^wt^ cells at a concentration of 4 mM NaHCO_3_ in DMEM cell culture media was chosen. The neutral cell culture media with a concentration of 44.05 mM NaHCO_3_ resulted in a pH_e_ of 7.7. Next, for murine CT26^wt^ and 4T1^wt^ cells, RPMI-1640 cell culture media supplemented with 10% FBS, 100 U ml^−1^ penicillin–streptomycin and NaHCO_3_ was selected. The cell culture media pH_e_ was again adjusted at 37 °C in a 5% CO_2_ atmosphere and determined after 5 h and 24 h. This resulted in a pH_e_ of 6.8 when applying a NaHCO_3_ concentration of 1 mM. Respectively a concentration of 23.81 mM (pH_e_ 7.5) or 1 mM NaHCO_3_ (pH_e_ 6.8) in RPMI-1640 was applied for all in vitro experiments with murine CT26^wt^ and 4T1^wt^ cells. For all in vitro experiments, a consistent terminology was chosen, independent whether cells were maintained in DMEM or RPMI cell culture media.

In one individual experiment (Fig. S2) 5x10^6^ MC38^wt^ tumor cells were cultured at 37 °C in a 5% CO_2_ atmosphere in acidic (pH = 6.8), intermediate (pH = 7.4) and neutral (pH = 7.7) media. The pH_e_ of the acidic cell media was adjusted by addition of HCl and the pH_e_ of neutral cell media was adjusted by addition of NaHCO_3_.

Neutral cell culture conditions (pH_e_ 7.7 for DMEM, pH_e_ 7.5 for RPMI) are abbreviated with N, whereas acidic cell culture conditions (pH_e_ 6.8) are abbreviated with A. In the presence of IFN-γ the abbreviations N^IFN−γ^ and A^IFN−γ^ were applied, respectively.

### siRNA transfection

Cells were seeded 24 h prior to transfection with 50 nM siRNAs (GE Healthcare Dharmacon, Lafayette, CO, USA) targeting murine STAT1 (M-058881–02) and human STAT1 (M-003543–01) or with a nontargeting siRNA (D-001206–13-20) using Lipofectamine 2000 in Opti-MEM. Twenty-four hours after transfection, cells were treated with IFN-γ (10 ng ml^−1^) and/or acidic media and harvested for Western blot, qRT-PCR or flow cytometry analyses 24 h later.

### RNA preparation, cDNA synthesis and quantitative real-time PCR (qRT-PCR)

Total RNA was isolated according to the manufacturer’s instructions (peqGOLD, VWR International, Radnor, PA, USA), including additional genomic DNA digestion with DNase I (peqGOLD, VWR International). The cDNA templates were synthesized using oligo(dT) primers (Eurofins, Ebersberg, Germany), nucleoside triphosphates (20 mM dNTPs, Amersham, UK), 5 × buffer (18064–014, Invitrogen, Carlsbad, CA, USA), SuperScript II Reverse Transcriptase (Invitrogen), β-mercaptoethanol (Carl Roth) and recombinant ribonuclease inhibitor (Promega, Madison, WI, USA). Target gene expression (primer list Table 1) was determined using SYBR® Green PCR Master Mix (Qiagen, Hilden, Germany) in a Light Cycler (Roche Diagnostics GmbH, Mannheim, Germany). The qRT-PCR conditions were as follows: initial activation at 95 °C for 15 s, followed by 40 amplification cycles at 95 °C for 15 s, 60 °C for 45 s and 72 °C for 30 s. Relative mRNA levels were normalized to the mean CT values of GAPDH, aldolase and β-actin.

**Table 1:** Primer sequences for qRT-PCR

Species	Primer	Sequence (5'- > 3')
murine	*Aldolase*	TGGGCCTTGACTTTCTCCTAT
		TGTTGATGGAGCAGCCTTAGT
	*β-actin*	CGGATGTCAACGTCACACTT
		GGCCAGGTCATCACTATTGG
	*Gapdh*	ACACATTGGGGGTAGGAACA
		AACTTTGGCATTGTGGAAGG
	*Pd-l1*	CGCCTGCAGATAGTTCCCAA
		ATCGTGACGTTGCTGCCATA
	*Stat1*	TTGACGACCCTAAGCGAACT
		TCAAATTCGGGGCCCACTAT
	*Mmp-2*	CACACCAGGTGAAGGATGTG
		AGGGCTGCATTGCAAATATC
	*Mmp-9*	CGTCGTGATCCCCACTTACT
		AACACACAGGGTTTGCCTTC
human	*Aldolase*	AATGTTCTGGCCCGTTATGC
		CCAGGTAGATGTGGTGGTCA
	*β-actin*	ACTCTTCCAGCCTTCCTTCC
		TCTCCTTCTGCATCCTGTCG
	*Gapdh*	CCAGAACATCATCCCTGCCT
		CCTGCTTCACCACCTTCTTG
	*Pd-l1*	GTGCCGACTACAAGCGAATT
		CTTGGAATTGGTGGTGGTGG
	*Stat1*	GTGGTACGAACTTCAGCAGC
		CATGAAAACGGATGGTGGCA

### Flow cytometry analysis

For the analysis of cell surface PD-L1 expression, cells were harvested after an incubation with trypsin, washed with PBS (Thermo Fisher Scientific) and passed through a 40 µm cell strainer with a snap cap (Corning, New York, United States). Single-cell suspensions were stained with a Zombie NIR™ Fixable Viability Kit (Biolegend, San Diego, CA, USA), BV605-CD274 (mouse, clone 10F.9G2, Biolegend) or BV605-CD274 (human, clone 29E.2A3, Biolegend) for 45 min at 4 °C, washed three times with PBS supplemented with 1% FCS and analyzed using a BD LSRFortessa flow cytometer and FlowJo Software (BD Bioscience, San Jose, CA, USA).

### Western blot assays

For immunoblotting, cells were lysed with RIPA buffer containing phosphatase II and III (50 µl, Sigma-Aldrich) and protease inhibitors (10 mM EDTA, oComplete, Roche). Protein lysates were sonicated (5 min), centrifuged (15 min) and separated on 10% SDS-PAGE gels. After transfer onto polyvinylidene difluoride (PVDF) membranes, washing with 0.05% Tween 20 in PBS (PBS-T) and blocking with intercept blocking buffer (LI-COR Bioscience, Lincoln, NE, USA), membranes were incubated with the primary antibodies. The primary antibodies STAT1 (9172), pSTAT1 (phospho Y701; 9167), elF4A1 (2490), elF4E (2067) and β-actin (4970) were purchased from Cell Signaling Technology. The primary anti-PD-L1 (AF1019) antibody was purchased from R&D Systems and anti-pSTAT1 (phospho Y701; 29045) was purchased from Abcam (Cambridge, UK). After washes with PBS-T, the membranes were incubated with either donkey anti-mouse (926–68072, IRDye® 680RD), anti-rabbit (926–68073, IRDye® 680RD and 925–32213, IRDye® 800CW) or anti-goat (926–68074, IRDye® 680RD) antibodies from LI-COR Bioscience and detected using the OdysseySA Infrared Imaging System (LI-COR Bioscience). For densitometry, signals were quantified using Image Studio Light Ver 5.2 software (LI-COR Bioscience). For the Western blot (WB) shown in Fig. 3A cells were lysed with lysis buffer containing 20 mM TRIS–HCl pH 7.5, 150 mM NaCl, 1% Triton X-100, 1 mM Na_2_EDTA, 1 mM EGTA, 1 mM β-glycerophosphate, 2 M urea and 1 × protease inhibitor cocktail (Roche) for 10 min on ice. Next, protein lysates were sonicated for 10 min using a bioruptor (Sonifier B-12 A), Laemmli buffer (0.5 M Tris/HCl pH 6.8, Trizma Base, Sigma Aldrich, T1503; HCL, Carl Roth, 6331.4; Glycerol 3 mL, Carl Roth, 3783.1; SDS 1 g,Carl Roth, CN30.2; Bromophenol blue 1.2 mg, Sigma Aldrich, B8026) was added, and the samples were separated by SDS-PAGE and transferred to a nitrocellulose membrane (Merck Millipore, IPFL00010).

### Experimental exogenous tumor models

Female C57BL/6 J, C57BL/6N (acidoCEST-MRI measurements) and BALB/c mice were purchased from Charles River Laboratories (Sulzfeld, Germany). B6.129S7-*Ifng*^*tm1Ts*^/J mice [33] were purchased from The Jackson Laboratory and bred in the animal facility in Tübingen. For direct comparison, C57BL/6 J mice bred in the same animal facility were used for the respective experiments. Animals were used at the age of 8 to 10 weeks and maintained in individually ventilated cages with standard rodent pellet food and water or 200 mM NaHCO_3_ water (3 days prior to cancer cell inoculation) [24] available ad libitum. Tumors were inoculated by *subcutaneous injection (s.c.)* of 500,000 MC38^wt^, 500,000 MC38^PD−L1−/−^, 100,000 CT26^wt^ or 100,000 CT26^PD−L1−/−^ cells in PBS into the right flank. B16-F10^wt^ tumors were inoculated by an *intracutaneous* (*i.c.)* injection of 125,000 cells in PBS into the right flank. 4T1^wt^ tumors were inoculated by an injection of 200,000 cells in PBS into the fourth mammary fat pad. Tumor outgrowth was measured in two dimensions, and the tumor volume was calculated using the formula (length*width*width)/2. Tumor-bearing mice were *intraperitoneally (i.p.)* injected with 200 µg of a PD-L1-blocking antibody (anti-PD-L1, clone 10F.9G2, Bio X Cell, West Lebanon, NH, USA) or IgG2b isotype control (clone LTF-2, Bio X Cell) every third day starting on day 4 (MC38^wt^, CT26^wt^, B16-F10^wt^) or day 5 (4T1^wt^) after the cancer cell inoculation. Mice were euthanized due to tumor burden (> 15 mm diameter, ulceration) and/or weight loss (> 20%), according to the local guidelines and regulations.

### Histology and immunohistochemistry (IHC)

Human metastatic melanoma tissue was fixed with formalin and embedded in paraffin. Tissue samples were cut into 5 μm sections and processed with hematoxylin eosin staining for the histological evaluation. For IHC, sections were cut into 3 µm sections (Leica CM1950). Staining was performed on an immunostainer (BOND-MAX, Leica Biosystems, Wetzlar, Germany) with mAbs against SOX10 (Cell Marque, Rocklin, CA, USA), CD3 (Leica Biosystems, Wetzlar, Germany), Ki67 (Agilent Technologies, Santa Clara, CA, USA) and PD-L1 (Cell Signaling Technology). Negative and positive controls were included. Images were analyzed using a Nikon Eclipse 80i microscope and a Digital Sight DS-U3 camera with NIS-Elements D software.

Murine tumors were fixed with 4% formalin and embedded in paraffin. For histology, 3–5 µm-thick sections were cut and stained with hematoxylin and eosin (H&E). H&E stained tumors were scanned with the Ventana DP200 (Roche, Basel, Switzerland) and processed with the Image Viewer MFC Application. Necrosis was evaluated in the H&E tumor scans, and the percentage of necrotic area per total tumor section area is reported (necrosis with an area smaller than 0.01 mm^2^ was not considered). Final image preparation was performed with Adobe Photoshop CS6. IHC was performed on selected samples with an automated immunostainer (Ventana Medical Systems, Tucson, AZ, USA) according to the manufacturer’s instructions for open procedures with slight modifications. The antibody panel used here included CD3 (Clone SP7; DCS Innovative Diagnostik-Systeme, Hamburg, Germany) and PD-L1 (Cell Signaling Technology, Frankfurt am Main, Germany). Appropriate positive and negative controls were used to confirm the adequacy of the staining. All images were acquired with an Axioskop 2 plus Zeiss microscope equipped with a Jenoptik (Laser Optik System, Jena, Germany) ProgRes C10 plus camera. Image analysis was performed with IMS Client Software.

### Fluorescence microscopy

For fluorescence microscopy, MC38^wt^ cells were grown and treated in chamber slides (354108, Falcon) and fixed with periodate-lysine-paraformaldehyde. After permeabilization with 0.5% Triton X 100 (T8787, Sigma Aldrich), cells were blocked with donkey serum (D9663, Sigma Aldrich) and then incubated with the respective primary antibodies. Paraffin-embedded MC38^wt^ tumor and human metastatic melanoma tissues were cut into 3–5 µm-thick sections, deparaffinized, unmasked with either citrate buffer (pH 6.0, Thermo Fisher Scientific) or EDTA buffer (pH 9.0, Thermo Fisher Scientific) and washed with distilled water, PBS (D8537, Sigma Aldrich) and PBS containing bovine serum albumin (900.009, Aurion) and Tween 20 (9127.1, Roth). Tissue sections were blocked with donkey serum, incubated with primary antibodies against PD-L1 (E1L3N, Cell Signaling Technology), Ki67 (SolA15, Thermo Fisher Scientific), Iba1 (EPR16588, Abcam), CD4 (4SM95, eBioscience), CD8a (4SM15, eBioscience), CD69 (GTX37447, GeneTex, Irvine, CA, USA), SOX10 (SOX10/1074, Abcam) and melanoma markers (HMB45 + M2-7C10 + M2-9E3 + T311, Abcam) and visualized by an incubation with Cy3 donkey anti-rabbit, Alexa Fluor 647 donkey anti-rat, Alexa Fluor 488 donkey anti-goat, Alexa Fluor 647 donkey anti-mouse or Alexa 488 donkey anti-mouse antibodies (all from Dianova, Hamburg, Germany). Nuclei were stained with DAPI (Sigma-Aldrich) or YO-PRO™-1 iodide (Molecular Probes, Inc., Eugene, OR, USA). Images were analyzed using a Zeiss LSM 800 and ZEN 2.3 software (blue edition). The PD-L1 fluorescence area and nuclei were quantified using ZEN Module Image Analysis.

### In vivo acidoCEST MRI measurement

Tumor pH_e_ was determined noninvasively in vivo by chemical exchange saturation transfer (CEST) MRI using iopamidol (Isovue®, Bracco Diagnostics, Milan, Italy) as an external contrast agent. In vivo MR imaging was performed on a preclinical 7 T BioSpec 70/30 MR scanner with a ^1^H volume coil (inner diameter: 86 mm; both Bruker BioSpin, Ettlingen, Germany). Experimental animals were anesthetized with 1.5% isoflurane in air (flow rate: 0.8 l/min). For contrast agent infusion, a catheter was placed into a lateral tail vein. Correct positioning of the animals was achieved with the aid of a short T_1_-weighted FLASH sequence (Table 2). MC38^wt^ tumors were localized using a standard axial 2D T_2_-weighted TurboRARE protocol (Table 2). Body temperature was monitored with a rectal probe and maintained at 35.0–37.8 °C with water-heating systems (Medres, Cologne, Germany). While respiration was monitored and the breathing rate was maintained between 30–50 breaths per minute, respiratory gating was not performed. AcidoCEST MRI was performed using a previously established CEST FISP acquisition protocol [34]. A detailed description of the CEST FISP sequence is provided in Table 2.

**Table 2:** Acquisition parameters for MR imaging

	**T1 FLASH**	**T2 TurboRARE**	**cestFISP**
TE	2.670 ms	33.580 ms	1.435 ms
TR	100 ms	5551.773 ms	2.870 ms
Flip angle	30.0°	-	30.0°
Spatial resolution	0.43 × 0.43	0.3 × 0.3 mm^2^	0.6 × 0.6 mm^2^
Matrix size	256 × 256	128 × 128	64 × 64
FOV	110 × 100 mm^2^	38.4 × 38.4 mm^2^	38.4 × 38.4 mm^2^
Slice thickness	1 mm	1 mm	1 mm
Slice orientation	Coronal	Axial	Axial
Number of averages	1	1	1
Number of repetitions	1	1	
Number of CEST spectra	-	-	4 preinjection, 6 postinjection
Saturation power	-	-	3 µT
Saturation pulses (pulse duration)	-	-	60 (100 ms)
Number of saturation frequencies	-	-	40
Saturation frequency range (increments), Hz	-	-	-30000 -4500 to -3600 (900) -3600 to 0 (600) 0 to 2100 (75) 2100 to 2700 (600) 2700 to 4500 (900)
Total acquisition time	12 s 800 ms	1 min 28 s 828 ms	44 min 22 s

The resulting spectra were fit using MATLAB (MATLAB R2017b, MathWorks, Inc., Natick, MA, USA) with a previously described Bloch fitting analysis method to generate pH_e_ maps [35]. Briefly, the preinjection and postinjection images were averaged at each saturation frequency and smoothed with a Gaussian spatial smoothing algorithm. The resulting averaged and smoothed preinjection image was subtracted from the postinjection image at each saturation frequency to correct for endogenous CEST signals. CEST spectra for each pixel were fitted with the Bloch-McConnell equations for the CEST effects from iopamidol at 4.2 and 5.6 ppm, as well as the water peak; pixels with insufficient contrast (defined as std_noise*2√2/mean_signal) were excluded. Additionally, pixels with pH_e_ values below pH 6.2 and above pH 7.4 were excluded from the analysis. The calculated pH_e_ map was overlaid on the anatomical reference, and data are reported as the average pH_e_ value derived from the sum of individual pixels.

### Transcriptome correlation analysis of cutaneous melanoma samples of patients

Transcriptomic data were retrieved via the ‘RTCGA’ package in R version 3.5.0. The results are based on data generated by TCGA Research Network: https://www.cancer.gov/tcga. The ‘expressions TCGA’ function was used to retrieve the mRNA expression profile of 368 cutaneous melanoma samples. RSEM values were log-transformed and a correlation analysis using the Pearson method was employed for the selected genes. Numbers represent the Pearson coefficients, statistically significant results (*p* < 0.05) are indicated by the presence of a colored circle. Scatter plots represent expression data across all 368 samples.

### Statistics

The experiments were not randomized. The investigators were not blinded to allocation during the experiments or outcome assessments. Statistical analysis and graph design were performed with GraphPad Prism (Version 7.03, GraphPad Software, Inc., USA). For the statistical analysis, Tukey’s multiple comparison test, Dunnett’s multiple comparison test, Sidak’s multiple comparison test, a two-tailed Student’s t-test or an unpaired, nonparametric Mann–Whitney test was applied. Statistically significant differences in the tumor pH_e_ determined using acidoCEST MRI between the NaHCO_3_-treated neutral^IFN−γ^ and untreated acidosis^IFN−γ^ group were determined using a one-tailed Mann–Whitney test, as only the increase in tumor pH_e_ upon NaHCO_3_ treatment was tested for statistical significance. *P*-values < 0.05 were considered significant (*).

## Results

### Acidosis promotes IFN-γ-induced PD-L1 expression by murine and human cancer cells

**Fig. 1:**
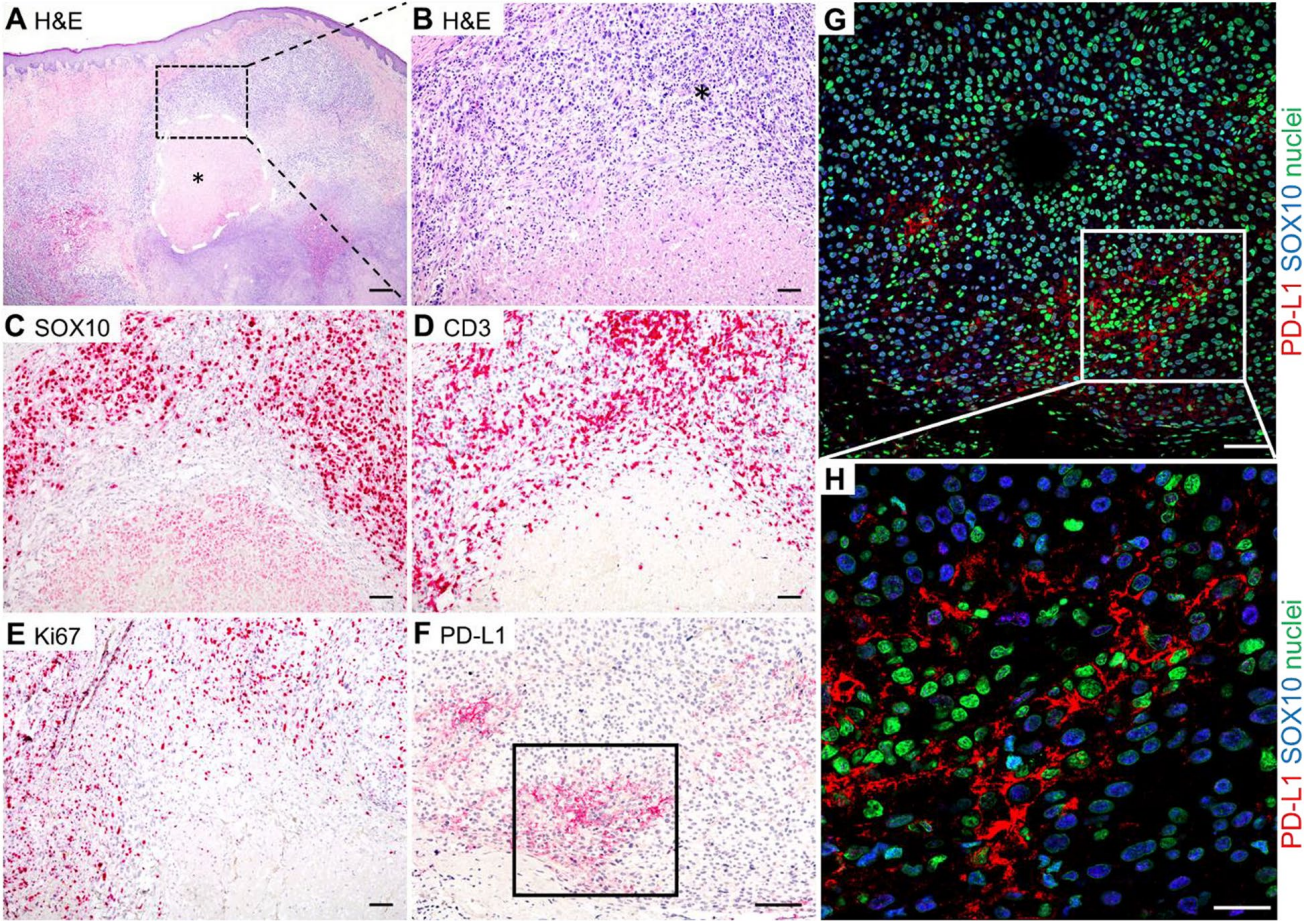
PD-L1 is expressed on melanoma cells located close to T cell-enriched tumor regions. H&E-stained human metastatic melanoma tissues (**A**, **B**) with necrotic tissue regions (black asterisk + encircled with white dashed lines). **B**-**F** Shows the magnification of the identical region of the tumor as indicated by the black rectangle (dashed lines) in (**A**). **B**-**F** In the viable melanoma tumor tissue (black asterisk), **C** SOX-10-expressing melanoma cells were discriminated from the (**D**) CD3 + T cell infiltrate. In addition, viable tumor regions were discriminated from necrotic tumor regions based on (**E**) Ki67 expression patterns. IHC showed that tumor regions with pronounced (**F**) PD-L1 expression (black rectangle) were located near the tumor immune cell infiltrate that was identified based on the cell morphology. **G** At higher magnification (white rectangle) it is visible that PD-L1 positive cells also express SOX-10. The immunofluorescence double staining of PD-L1 and SOX10 (H) of the serial section (**F**) shows the same region of the tumor (black rectangle) with PD-L1 positive cells surrounding an immune cell infiltrate. Scale bars: 500 μm (**A**), 100 μm (**B**-**F**), 50 μm (**H**), 20 μm (**G**)

IFN-γ derived from activated immune cells (T cells, NK cells and macrophages) located close to acidic tumor regions may promote the expression of the PD-L1 gene in cancer cells in these regions. Consistent with these data, we observed the phenomenon of PD-L1 expression on cancer cells near the T cell-enriched tumor regions in human melanoma metastases (Fig. 1A-H). Histopathological analysis of viable tumor tissue (H&E staining and Ki67 IHC staining) revealed increased PD-L1 expression in SOX10-positive melanoma cells located exclusively near CD3^+^ T cell-enriched melanoma regions, close to necrotic tumor areas (Fig. 1A-H). Thus, we hypothesized that tumor-infiltrating, IFN-γ-secreting immune cells induce PD-L1 expression on cancer cells and thereby mediate immune escape.

**Fig. 2:**
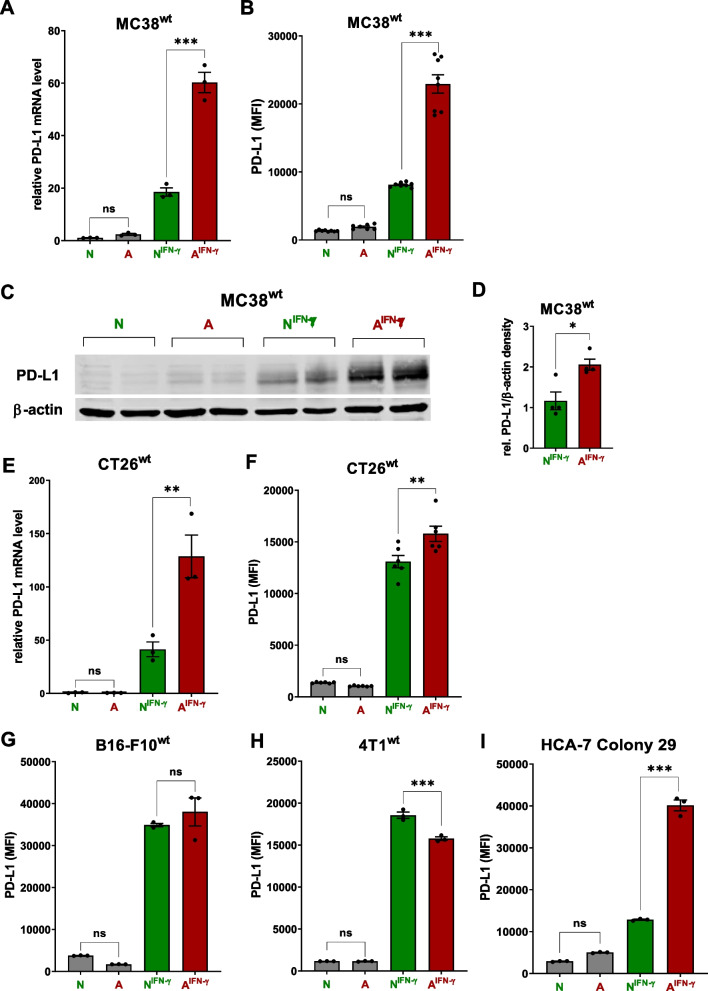
*A*^*IFN−γ*^ induces PD-L1 expression in anti-PD-L1 mAbs therapy-responsive murine cell lines while PD-L1 expression is not significantly enhanced in those that are non-responsive. **A** Relative *Pdl1* mRNA expression normalized to *Gapdh*, Aldolase and β-actin (*n* = 3, statistics: Tukey’s multiple comparison test), **B** PD-L1 mean fluorescence intensity (MFI) measured using flow cytometry (pooled data from 3 experiments, *n* = 8, statistics: Tukey’s multiple comparison test) and (**C**, **D**) Western blot analysis and densitometry of PD-L1 and β-actin levels (pooled data from 2 experiments, *n* = 4, statistics: two-tailed Mann–Whitney test) in MC38^wt^ cells treated with acidic media and/or IFN-γ (10 ng ml^−1^) for 72 h. Data are presented as the means ± SEM. N = neutral media, A = acidic media, N^IFN−γ^ = neutral media plus IFN-γ, A^IFN−γ^ = acidic media plus IFN-γ. **E** Relative *Pdl1* mRNA expression normalized to Gapdh, Aldolase and β-actin (*n* = 3) and (**F**) PD-L1 MFI measured using flow cytometry (pooled data from 2 experiments, *n* = 6) in murine anti-PD-L1-responsive CT26^wt^ cells following treatment with acidic media and/or IFN-γ (10 ng ml^−1^) for 72 h. PD-L1 MFI of the (**G**) nonresponsive murine B16-F10^wt^ and (**H**) 4T1^wt^ cell lines (*n* = 3) and (**I**) human HCA7 colony 29 cells (*n* = 3) treated with acidic media and/or IFN-γ (10 ng ml^−1^) for 72 h. Data are presented as the means ± SEM. Statistics: Tukey’s multiple comparison test. N = neutral media, A = acidic media, N^IFN−γ^ = neutral media plus IFN-γ, A^IFN−γ^ = acidic media plus IFN-γ

We used either neutral (N, pH_e_ = 7.5 (RPMI), 7.7 (DMEM)) or acidic (A, pH_e_ = 6.8 (RPMI and DMEM) culture conditions in the presence or absence of IFN-γ and studied PD-L1 expression in cancer cells to investigate the role of acidosis. Acidic conditions were confirmed by determining the mRNA levels of acidosis-associated surrogate markers such as MMP-2 and MMP-9 [36] and enhanced proliferation [37] of MC38^wt^ cells (Fig. S2A and B)*.* An acidic pH_e_ was generated by lowering the NaHCO_3_ concentration in cell culture media. We first investigated anti-PD-L1-responsive MC38^wt^ murine colon carcinoma cells to study the effect of basal and induced PD-L1 expression mediated by IFN-γ-secreting immune cells and the acidic TME. IFN-γ treatment under neutral conditions (N^IFN−γ^) induced *Pdl1* mRNA expression, which was further increased under acidic and IFN-γ conditions (A^IFN−γ^). Elevated *Pdl1* mRNA expression (Fig. 2A) caused a significant, more than two-fold increase in membrane (Fig. 2B) and total PD-L1 protein levels (Fig. 2C and D), which was also confirmed by fluorescence microscopy (Fig. S3).

Similar to MC38^wt^ cells, the addition of IFN-γ to acidic culture media increased *Pdl1* mRNA (Fig. 2E) and PD-L1 cell surface expression (Fig. 2F) but not total protein expression in murine CT26^wt^ colon carcinoma cells (Fig. S4B and C) with low basal PD-L1 expression (PD-L1^low^, Fig. S5A-C).

Next, we asked whether cancer cells that are nonresponsive to anti-PD-L1 therapy, such as B16-F10^wt^ melanoma and 4T1^wt^ mammary carcinoma cells [38], differ in their PD-L1 expression pattern. In sharp contrast to the responsive MC38^wt^ and CT26^wt^ cancer cells, no acidosis-induced increase in cell surface PD-L1 expression was observed upon IFN-γ treatment in B16-F10^wt^ and 4T1^wt^ cancer cells (Fig. 2G, H).

Moreover, we screened five human cancer cell lines, colon adenocarcinoma (HCA-7 colony 29), breast cancer (MCF-7), glioma (U-87 MG), malignant melanoma (SK-MEL-28), pancreatic adenocarcinoma (MIA PaCa-2) to explore whether the acidosis- and IFN-γ-induced increase in PD-L1 expression represents a conserved tumor immune escape mechanism (Fig. 2I, S6A). Thus, we observed a significant A^IFN−γ^-induced increase in PD-L1 expression in the HCA-7 colony 29 (Fig. 2I), MCF-7 and U-87 MG (Fig. S6A) cancer cell lines compared to neutral conditions. Moreover, the addition of IFN-γ to acidic cell culture media further increased PD-L1 mRNA (Fig. S6B) and total protein levels in HCA-7 colony 29 cells (Fig. S6C) suggesting responsiveness to therapeutic blockade of the PD-1/PD-L1 axis.

These results, therefore, indicate that the combination of IFN-γ and acidosis increases the expression of the PD-L1 mRNA, cell surface, and total proteins in various murine and human cancer cells.

### Acidosis promotes IFN-γ-induced PD-L1 gene expression by increasing the phosphorylation of STAT1

**Fig. 3:**
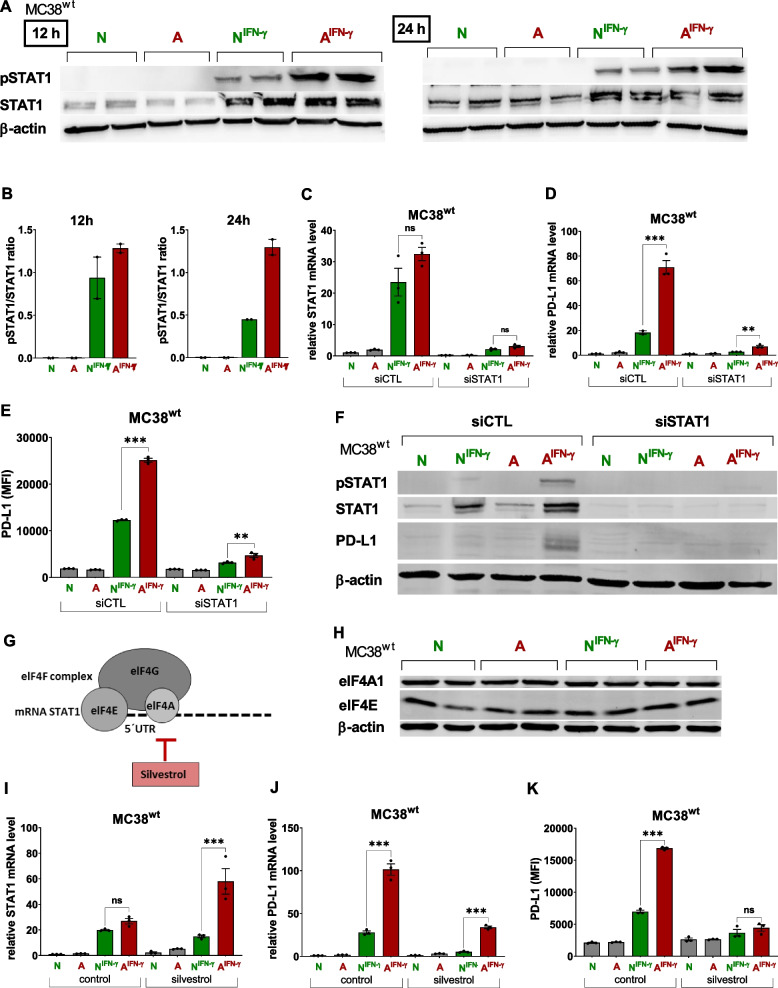
STAT1 is required for A^IFN−γ^-induced PD-L1 expression in cancer cells and elF4F inhibition blocks A^IFN−γ^-mediated PD-L1 expression in cancer cells. **A** Analysis of pSTAT1, STAT1 and β-actin protein levels in MC38^wt^ cells treated with acidic media and/or IFN-γ (10 ng ml^−1^) for 12 h and 24 h as determined using Western blotting (**B**) Densitometry of pSTAT1/STAT1 Western blotting of A. **C**-**F** MC38^wt^ cells were transfected with a control (siCTL) or *Stat1*-specific siRNA and treated for 24 h with acidic media ± IFN-γ (10 ng ml^−1^) before the assessment of relative (**C**) *Stat1* and (**D**) *Pdl1* mRNA expression normalized to *Gapdh*, *Aldolase* and *β-actin* (*n* = 3) and (**E**) cell surface PD-L1 expression (*n* = 3) using qRT-PCR and flow cytometry, respectively. (**A**, **H**) The two signals per condition represent two independent samples (*n* = 2); two independent experiments showed similar results. Statistics: Tukey’s multiple comparison test. **F** Western blot analyses of pSTAT1, STAT1, PD-L1 and β-actin levels were performed to determine the STAT1 knockdown efficiency (*n* = 2). Data are presented as the means ± SEM. N = neutral media, A = acidic media, N^IFN−γ^ = neutral media plus IFN-γ, A^IFN−γ^ = acidic media plus IFN-γ. **G** Schematic representation of the eukaryotic translation initiation complex elF4F composed of elF4G, elF4E and elF4A (inhibited by silvestrol) bound to the 5’UTR of the *Stat1* mRNA. **H** Western blot analysis of elF4A1, elF4E and β-actin levels in MC38^wt^ cells treated with acidic media and/or IFN-γ (10 ng ml^−1^) for 72 h (*n* = 2). Relative (**I**) *Stat1* and (**J**) *Pdl1* mRNA expression normalized to *Gapdh*, *Aldolase* and *β-actin* (*n* = 3) and (**K**) cell surface PD-L1 expression (*n* = 3) were determined in MC38^wt^ cells treated with acidic media and/or IFN-γ (10 ng ml^−1^) in the presence of DMSO (control) or silvestrol (30 nM) for 24 h. Data are presented as the means ± SEM. Statistics: Tukey’s multiple comparison test. N = neutral media, A = acidic media, N^IFN−γ^ = neutral media plus IFN-γ, A^IFN−γ^ = acidic media plus IFN-γ

IFN-γ increased STAT1 protein levels in MC38^wt^ (Fig. 3A and B) and CT26^wt^ cells (Fig. S4B and C) to the same degree under A^IFN−γ^ and N^IFN−γ^ conditions without altering *Stat1* mRNA levels (Fig. 3C and S4A). Interestingly, acidosis strongly increased IFN-γ-induced STAT1 phosphorylation in MC38^wt^ cells (Fig. 3A and B) but not in CT26^wt^ cells (Fig. S4B). CT26^wt^ cells revealed only a moderate induction of cell surface PD-L1 expression upon treatment with IFN-γ under acidic conditions (Fig. 2F). Similarly, in human HCA-7 colony 29 cells, the IFN-γ-induced increase in PD-L1 expression under acidic culture conditions (Fig. 2I and S6C) was associated with increased phosphorylation but not the expression of STAT1 (Fig. S6C). Thus, our data indicate that acidosis increases IFN-γ-mediated STAT1 phosphorylation and PD-L1 expression.

We next conducted siRNA-mediated STAT1 knockdown experiments in murine MC38^wt^ and human HCA-7 colony 29 cancer cells to further elucidate the mechanism of A^IFN−γ^-induced PD-L1 expression. Knockdown of STAT1 in MC38^wt^ cells prevented the inducibility of the *STAT1* and *PD-L1* mRNAs (Fig. 3C and D), as well as membranous (Fig. 3E) and total PD-L1 protein expression (Fig. 3F). In a similar manner knockdown of STAT1 in HCA-7 colony 29 cancer cells prevented from PD-L1 protein synthesis (Fig. S6D).

### Acidosis- and IFN-γ-induced PD-L1 expression depends on elF4F

We investigated the role of translational regulation in IFN-γ-induced STAT1 and PD-L1 expression under acidic conditions. According to recent studies, silvestrol, an inhibitor of the helicase activity of eukaryotic initiation factor 4A, prevents translation of the *Stat1* mRNA [39] (Fig. 3G) and might therefore represent a potential therapeutic option to avoid tumor immune escape via the STAT1-PD-L1 axis. In our experimental setting, acidosis showed no effect on the expression of the elF4F complex components elFA1 and elF4E in MC38^wt^ cancer cells (Fig. 3H).

Silvestrol upregulated Stat1 mRNA expression under A^IFN−γ^ conditions (Fig. 3I). This is consistent with the results reported by Cerezo et al*.* that showed enhanced Stat1 mRNA levels after exposure to IFN-γ and silvestrol [40]. The increase in Stat1 mRNA levels after stimulation by A^IFN−γ^ and inhibition by silvestrol results from the accumulation of non-translated Stat1 mRNA [41]. As shown in Fig. 3D and E, A^IFN−γ^ -induced PD-L1 mRNA and cell surface expression in MC38^wt^ cells.

Importantly, silvestrol treatment inhibited A^IFN−γ^-induced PD-L1 mRNA and cell surface upregulation in MC38^wt^ cells (Fig. 3J and K). The PD-L1 mRNA and cell surface expression were similar to normal pH condition (Fig. 3J and K). Thus, our results support the key role of STAT-1 activation for A^IFN−γ^ -mediated PD-L1 expression.

Following these studies, we asked whether our in vitro findings were also valid in vivo. Thus, the cancer cell lines with different basal, non-stimulated PD-L1 expression patterns studied in vitro (Fig. S5A) were examined in vivo. MC38^wt^ (PD-L1^high^) and CT26^wt^ (PD-L1^low^) carcinomas are responsive to anti-PD-L1 mAb therapy, whereas B16-F10^wt^ (PD-L1^high^) and 4T1^wt^ (PD-L1^low^) carcinomas show low-/nonresponsive behavior (Fig. S5A) [42], suggesting that the anti-PD-L1 mAb therapy response is independent of basal PD-L1 expression on cancer cells.

### NaHCO_3_-treatment neutralizes the extracellular tumor pH_e_

**Fig. 4:**
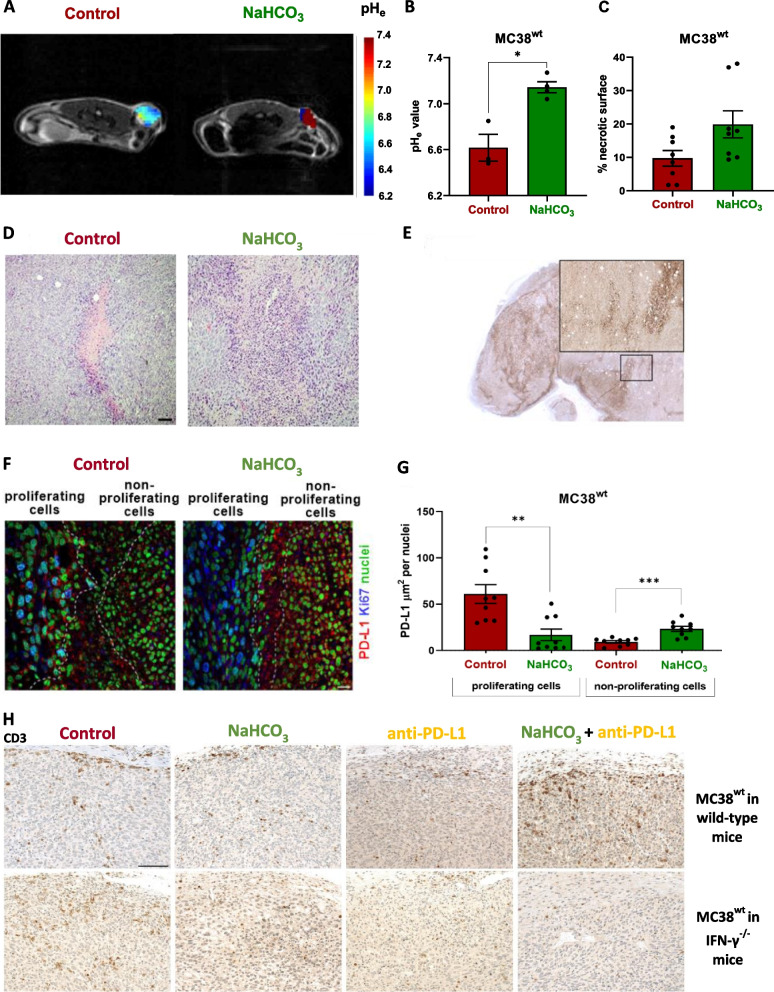
Tumor acidosis increases PD-L1 expression on cancer cells and alleviates the immigration of CD3^+^ T cells. **A** Representative pH_e_ maps overlaid on T_2_-weighted axial MRI images of MC38^wt^ tumor-bearing mice injected with iopamidol (*i.v.*) at day 10 after the cancer cell injection. Mice with intact IFN-γ signaling received either NaHCO_3_-enriched water three days prior to cancer cell injection or regular drinking water (Control). Individual images of the tumors are shown in Fig. S7. **B** Average pH_e_ across the whole tumor. The NaHCO_3_ treatment significantly increased the tumor pH_e_ measured using acidoCEST MRI (*n* = 3–4 animals per group; 2 mice were excluded from the quantitative analysis because the measured pH_e_-values were out of the calibration range, see Fig. S7, statistics: one-tailed Mann–Whitney test). **C** The percentage of necrosis in MC38^wt^ tumors from the NaHCO_3_ and Control groups was quantified using H&E staining, as shown in Fig. S8. **D** Representative H&E staining of MC38^wt^ tumors isolated from experimental mice on day 18 after control or NaHCO_3_ treatment. H&E staining show the well-delimited necrotic area in the MC38^wt^ tumor of a control treated mouse (center, pink area). In contrast, within the MC38^wt^ tumor of a NaHCO_3_ treated mouse the necrosis is diffuse surrounded by areas of hypoxia reflected by abundant pyknotic cells (hyperchromatic; magnitude: 10x; scalebar: 100 µm). **E** Representative PD-L1 IHC of a MC38^wt^ tumor isolated from experimental mice on day 18 after control treatment. (magnitude: 12.5x, scalebar 2 mm; insert: 50x; scalebar 500 µm). **F** Representative fluorescence microscopy images of PD-L1, the proliferation marker Ki67 in MC38^wt^ tumors isolated on day 18 after treatment with NaHCO_3_ and control groups. **G** PD-L1 expression was quantified in proliferating and non-proliferating cells from MC38^wt^ tumor regions. Statistics: two-tailed Mann–Whitney test. **H** Representative CD3 immunohistochemistry of *s.c.* MC38^wt^ tumors derived from wild-type C57BL/6 J (day 18) or IFN-γ^−/−^ mice (day 17) after the four different treatment conditions. Animals received regular drinking water (control), NaHCO_3_ in water (NaHCO_3_), anti-PD-L1 mAb or NaHCO_3_ & anti-PD-L1 mAb. N = 3 – 4 representative tumors of each experimental group were subjected to CD3 staining. (magnitude: 400x; scalebar: 100 µm)

We used NaHCO_3_ to neutralize the tumor pH_e_ (NaHCO_3_) in vivo to study the effect of the tumor pH_e_ on cancer PD-L1 expression and immune cell recruitment in mice with intact IFN-γ secretion (Control) [24]. Noninvasive in vivo acidoCEST MRI measurements confirmed a significant increase in the MC38^wt^ tumor pH_e_ upon NaHCO_3_ treatment (avg. pH_e_ 7.15) compared to MC38^wt^ tumors from mice receiving regular drinking water (avg. pH_e_ 6.52, control, *P < 0.05; Fig. 4A, B and S7). Furthermore, we identified acidic clusters within MC38^wt^ tumors, indicating intratumor heterogeneity (Fig. 4A and S7).

### Tumor acidosis increases PD-L1 expression in cancer cells

Here we aimed to analyze, whether acidosis-induced PD-L1 expression on cancer cells might represent an immune escape mechanism associated with tumor progression in vivo. MC38^wt^ tumors from untreated control mice exhibited significantly less necrosis than tumors from the NaHCO_3_ solo treatment (Fig. 4C and S8). The NaHCO_3_ solo treatment led to an increased percentage of necrotic surface, and this was positively correlated with the rise in pH_e_, as determined by acidoCEST-MRI (Fig. 4A-C).

In Fig. 4D, H&E staining of an MC38^wt^ tumor is represented, comparing control-treated and NaHCO_3_-treated experimental mice. Control treatment resulted in a well-defined necrotic area at the center of the MC38^wt^ tumor (highlighted in pink). In contrast, NaHCO_3_ treatment induced more extensive and diffuse necrosis within the MC38^wt^ tumor, which was surrounded by regions of hypoxia, evident from the presence of numerous pyknotic cells (Fig. 4D). The representative PD-L1 IHC image in Fig. 4E reveals that nearly all MC38^wt^ tumor cells displayed positive staining for PD-L1. Notably, PD-L1 expression was heterogeneous, with some cells showing weak expression while clusters of others exhibited intense PD-L1 expression. The regions with the highest PD-L1 expression were primarily located within the necrotic areas (Fig. 4E).

Within MC38^wt^ tumors of untreated mice we determined an increased PD-L1 expression on proliferating Ki67^+^ cells compared to MC38^wt^ tumors of NaHCO_3_-treated mice (Fig. 4F and G). In contrast, MC38^wt^ tumors derived from NaHCO_3_-treated mice exhibited increased PD-L1 expression on non-proliferating Ki67^−^ cells (Fig. 4F and G).

Treatment of MC38^wt^ tumor-bearing mice which underwent NaHCO_3_ and anti-PD-L1 (NaHCO_3_ + anti-PD-L1) treatment revealed an increased CD3^+^ T cell infiltration in the tumor periphery with few scattered CD3^+^ T cells within the TME (Fig. 4H). Pronounced CD3^+^ T cell infiltration was also observed in CT26^wt^ and 4T1^wt^ tumors from the NaHCO_3_ + anti-PD-L1-treated group (Fig. S9). In B16-F10^wt^ tumors of NaHCO_3_-treated experimental mice, we determined a pronounced infiltrate of tumor-adjacent CD3^+^ T cells but not in the combined NaHCO_3_- and anti-PD-L1-treatment group (Fig. S9). In 4T1^wt^ tumors of NaHCO_3_-treated experimental mice we observed a CD3^+^ T cell infiltrate at the tumor margins of all experimental groups. Nevertheless, the strongest accumulation of CD3^+^ T cells was found in 4T1^wt^ tumors of experimental mice upon combined treatment with NaHCO_3_ and anti-PD-L1 (Fig. S9). As expected MC38^wt^ tumors of tumor-bearing IFN-γ^−/−^ mice exhibited no enhancement of CD3^+^ T cell accumulation as a consequence of NaHCO_3_ or anti-PD-L1 treatment (Fig. 4H).

**Fig. 5:**
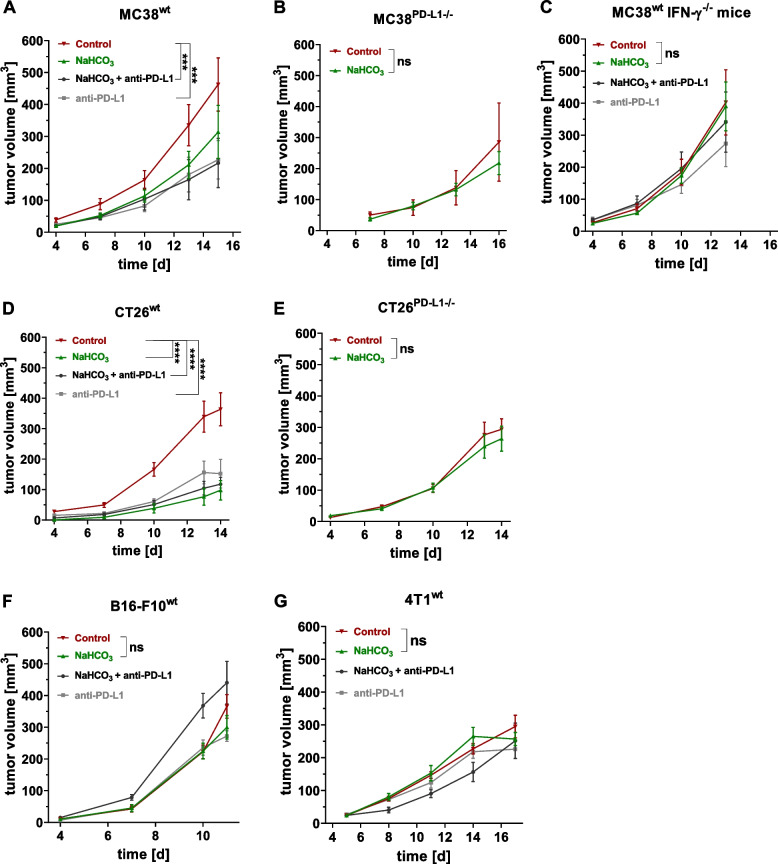
Tumor growth upon a combinatory or mono-treatment with NaHCO_3_ and anti-PD-L1 in anti-PD-L1 responsive and nonresponsive tumor models. **A** Tumor volumes of MC38^wt^ (*n* = 8 animals per group), **B** MC38^PD−L1−/−^ (*n* = 4–5 animals per group), **C** IFN-γ knockout mice (neutral and acidosis, *n* = 7–8 animals per group). **D** CT26^wt^ (*n* = 7–8 animals per group), and **E** CT26^PD−L1−/−^ (*n* = 8 animals per group) tumor volumes  of mice treated with NaHCO_3_ and/or anti-PD-L1 mAb. **F** Tumor volumes of B16-F10^wt^ (*n* = 5–8 animals per group) and (**G**) 4T1^wt^ tumors (*n* = 5–8 animals per group) growing in mice treated with NaHCO_3_ and/or anti-PD-L1 mAb. Treatment with NaHCO_3_-enriched water (200 mM, NaHCO_3_) started three days prior to cancer cell inoculation; anti-PD-L1 mAb (200 µg per mouse) was administered every third day starting on day 4 (MC38^wt^, CT26^wt^, B16-F10^wt^) or day 5 (4T1^wt^) after the cancer cell inoculation. Tumors from mice with intact IFN-γ signaling that received regular drinking water developed an acidic tumor pH_e_ (Control). Data are presented as the means ± SEM. Statistics: Tukey’s multiple comparison test (**A**, **C**, **D**, and **F**) and Sidak’s multiple comparison test (**B** and **E**)

We used IFN-γ^−/−^ mice to study whether IFN-γ secretion by activated tumor-infiltrating immune cells promotes PD-L1 expression on the membrane of MC38^wt^ cells in the untreated experimental group in vivo [24]. In contrast to wild-type mice (Fig. 5A), tumor pH_e_ neutralization in IFN-γ^−/−^ mice (NaHCO_3_) failed to slow tumor growth (Fig. 5C). MC38^wt^ tumors growing in IFN-γ^−/−^ mice exhibited only a moderate anti-PD-L1 response (Fig. 5C).

Thus, these results support the hypothesis that acidosis-induced PD-L1 expression on cancer cells represents an unknown mechanism of immune evasion, which anti-PD-L1 antibodies might effectively target.

### In vivo consequences of acidosis^IFN−γ^-mediated PD-L1 expression on cancer cells

In addition, we analyzed the effect of acidosis and IFN-γ induced PD-L1 expression on the in vivo growth of different cancer cell types. MC38^wt^ and CT26^wt^ tumors displayed decreased tumor growth in vivo upon NaHCO_3_ treatment compared to the control (Fig. 5A and D). In addition, the reduction in tumor volume was more pronounced in CT26^wt^ tumors with low basal (PD-L1^low^) and only weakly inducible PD-L1 expression (Fig. 5D) than in MC38^wt^ tumors with high basal (PD-L1^high^) and more pronounced inducible PD-L1 expression (Fig. 5A). As expected, both MC38^wt^ and CT26^wt^ tumors responded to anti-PD-L1 treatment. They showed a significant reduction in tumor volume [12]. Neither MC38^wt^ nor CT26^wt^ tumors in the NaHCO_3_ + anti-PD-L1 treatment group exhibited an additive therapeutic effect (Fig. 5A and D).

PD-L1-deficient MC38^PD−L1−/−^ and CT26^PD−L1−/−^ (Fig. 5B and E) tumor-bearing mice were treated with NaHCO_3_ or received regular drinking water (control) to prove that acidosis-induced PD-L1 expression on cancer cells mediates immune escape. Compared to MC38^wt^ and CT26^wt^ tumors, PD-L1-deficient tumors from the untreated control group exhibited lower tumor volumes (Fig. 5B and E). Furthermore, regardless of the tumor pH_e_, PD-L1-deficient MC38^PD−L1−/−^ and CT26^PD−L1−/−^ tumors exhibited similar tumor growth in mice (NaHCO_3_ vs. control, Fig. 5B and E). Thus, inducible cancer cell PD-L1 expression represents an essential feature associated with immune escape.

B16-F10^wt^ and 4T1^wt^ tumors, which were not susceptible to A^IFN−γ^-mediated PD-L1 induction in vitro (Fig. 2G and H), did not show a significant reduction in tumor volume in the NaHCO_3_ -treated experimental group compared to the untreated control group (Fig. 5F and G). As expected, B16-F10^wt^ and 4T1^wt^ tumors showed no or only a slight reduction in tumor volume upon anti-PD-L1 treatment (Fig. 5F and G).

### Correlation analysis of cutaneous melanoma samples of patients

Finally, we have conducted correlation analysis of the transcriptomic data of 368 cutaneous melanoma samples from patients and found a significant positive correlation of PD-L1 and IFN-γ (*r* = 0.737; *p* < 0.001), PD-L1 and STAT-1 (*r* = 0.778; *p* < 0.001), PD-L1 and IFN-γ driven CXCL10 (*r* = 0.721; *p* < 0.001), PD-L1 and the IFN-γ driven CXCL9 (*r* = 0.733; *p* < 0.001) and PD-L1 and acidosis driven MMP9 (*r* = 0.338; *p* < 0.001; Fig. S10).

In summary, our results indicate that acidosis and IFN-γ-mediated PD-L1 expression on cancer cells represents a previously undescribed novel immune escape mechanism that can be targeted by therapeutic blockade of the PD-L1/PD-1 axis. Thus, we propose that acidosis and IFN-γ-inducible PD-L1 expression represents an additional biomarker for the therapeutic response than tumor PD-L1 expression or T cell homing patterns.

## Discussion

The present study explored whether extracellular tumor acidosis and IFN-γ-inducible PD-L1 expression represent a mechanism of immune escape and, therefore, a novel biomarker for the therapeutic response.

We show that acidosis further increases IFN-γ-mediated PD-L1 expression on the surface of various human and murine cancer cell lines. The increase in IFN-γ-mediated PD-L1 expression by acidosis relies on STAT1 gene expression and translation of *Stat1* mRNA by the eukaryotic translation initiation factor elF4F, whose subunit, the RNA helicase eIF4A, binds and unwinds a selective subset of mRNAs, including *Stat1* [40]. Therefore, acidosis enhanced IFN-γ-induced STAT1 activation via phosphorylation, implying a feed-forward mechanism of acidosis in IFN-γ-induced PD-L1 expression and immune escape.

Notably, STAT1 knockdown and the use of the eIF4A subunit inhibitor silvestrol revealed a predominant role of STAT1 translation and phosphorylation in the acidosis-mediated increase in IFN-γ-induced PD-L1 expression. Our findings suggest a hitherto unknown link between acidosis- and IFN-γ-mediated PD-L1 expression that might represent a mechanism of immune escape. The proposed mechanism mediated by elF4A, an essential regulator of STAT1 and therefore PD-L1 expression [40, 43, 44], upon IFN-γ stimulation and increased STAT1 phosphorylation under acidic conditions opens a window for therapeutic interventions to alleviate immune escape of cancer cells by targeting PD-1/PD-L1 signaling. There is some evidence that PD-L1 expression is mainly regulated by activated STAT1 [45]. However, we cannot exclude the possibility that other STAT members including STAT3 may impact membranous PD-L1 expression upon dual stimulation with acidosis and IFN-γ. Kwon et al. reported an increased STAT3 phosphorylation under acidic media conditions, which was associated with elevated PD-L1 expression in MDA-MB-231 breast cancer cells. However, it is worth noting that in the referred study co-stimulation with acidic media and IFN-γ has not been investigated.

Tumor acidosis has been targeted preclinically by a NaHCO_3_ treatment, which increases the tumor pH_e_, and can be measured with microelectrodes [24] or noninvasively using ^31^P magnetic resonance spectroscopy (MRS) [46] and acidoCEST MRI [24, 46, 47].

The relevance of acidification of the TME has been recently reported by Cappellesso et al*.* which have experimentally proven that inhibition of the bicarbonate transporter SLC4A4 in pancreatic adenocarcinoma cells mitigated acidosis within the TME due to bicarbonate accumulation in the extracellular space. Inhibition of SLC4A4 in combination with immune checkpoint inhibitor treatment was applicable to overcome immunotherapy resistance and to prolong the survival of pancreatic adenocarcinoma bearing mice [48].

Counteracting the acidification of the TME reduced MC38^wt^ colon adenocarcinoma tumor volumes and increased necrotic regions. Consistent with our findings, Faes et al*.* reported a significant reduction in the MC38^wt^ tumor volume and increased necrosis upon NaHCO_3_ treatment [49]. Interestingly, in lung adenocarcinoma, necrosis significantly correlated with PD-L1 expression [50]. Tumor acidosis has been reported to impair IFN-γ secretion by activated cytotoxic CD8^+^ T cells [24] and NK cells [51]. Moreover, acidosis suppresses the release of TNF from macrophages [52] and monocytes [44]. Thus, the NaHCO_3_ treatment restores the secretion of cytokines [51], leading to increased PD-L1 expression in immune cells [53]. Since NaHCO_3_ proved intolerable for patients, several other pharmaceutical compounds targeting TME acidification are currently being tested in clinical trials. These compounds target members of the monocarboxylate transporter [54], sodium hydrogen antiporter [55], and carbonic anhydrase families [56]. Counteracting the acidification of the TME or selective inhibition of elF4A might open a new window for therapeutic intervention. Notably, since the translation of several oncogenes is also eIF4A-dependent, eiF4A inhibitors, such as silvestrol and rocaglaol, have been proposed as promising anticancer agents [57].

We show that the efficacy of NaHCO_3_ and anti-PD-L1 treatment is independent of basal tumor PD-L1 expression and that the induction of PD-L1 expression in response to tumor acidosis or IFN-γ secretion in the TME might represent an additional biomarker for prediction of a potential therapeutic response. Interestingly, CT26^wt^ tumors with a modest increase in PD-L1 expression upon stimulation with the combination of IFN-γ and acidosis responded better to NaHCO_3_-mediated pH_e_ neutralization than MC38^wt^ tumors. According to several studies, PD-L1 expression, either on tumor or immune cells, mediates tumor immune escape, rendering total tumor PD-L1 expression more reliable biomarker for predicting the response to anti-PD-L1 therapy [11–13, 58]. Our results suggest that the induction of PD-L1 expression by tumor acidosis and IFN-γ secretion within the TME represents a targetable mechanism of immune escape and determines the response. The two tumor models that did not respond to anti-PD-L1 therapy, B16-F10^wt^ and 4T1^wt^, did not respond to NaHCO_3_-mediated pH_e_ neutralization. Our observation concurs with a study by Pilon-Thomas *et al.* showing the lack of therapeutic response in NaHCO_3_-treated B16 tumor-bearing mice [24]. Our results also strongly correlate with recent literature demonstrating that blocking the PD-1/PD-L1 axis with an anti-PD-1 antibody leads to a substantial reduction in tumor growth of MC38 or CT26 tumor bearing mice [59–61]. In contrast to our results, Williams et al*.* have shown that CD8^+^ T cells efficiently inhibit the growth of IFN-γ receptor 2- or JAK1-deficient B16-F10 tumors which are not sensitive to IFN-γ signaling and subsequent PD-L1 upregulation [17].

Several studies suggest a combined immunohistological assessment of PD-L1 expression and tumor-infiltrating lymphocytes to determine IFN-γ-induced PD-L1 expression in the tumor immune microenvironment [62–64]. This strategy is promising, as it considers immune cell activity, shaped by tumor acidosis. The TME, the infiltration and activation of T cells [24, 65, 66] and macrophage polarization [67] are altered by tumor acidosis. In prostate cancer, tumor acidosis drives macrophages into a protumor phenotype [67]. Furthermore, a low tumor pH_e_ drives immune cells such as CD8^+^ T cells into an anergic state characterized by reduced cytolytic activity and cytokine secretion [66]. In contrast, tumor pH_e_ neutralization improves the response to anti-CTLA-4 and anti-PD-1 mAb immunotherapies [24].

Our correlation analysis of the transcriptomic data of 368 cutaneous melanoma samples revealed a positive correlation of PD-L1 to IFN-γ, STAT-1, IFN-γ driven CXCL10 and CXCL9 as well as to acidosis driven MMP9.

For validation of our proposed novel PD-L1 biomarker approach in a human cohort, we aim to take tumor biopsies to generate tumor cell suspensions. These tumor cells will be stimulated with IFN-gamma upon acidic and neutral culture conditions and changes in membranous PD-L1 expression analyzed via flow cytometry analysis (before and after stimulation). An additional acidosis related increase in PD-L1 expression upon stimulation with IFN-gamma would predict sensitivity to therapeutic blockade of the PD-1/PD-L1 axis.

## Conclusions

In conclusion, our data indicate that combining IFN-γ and acidosis-inducible PD-L1 expression on cancer cells represents a tumor immune escape mechanism. To our knowledge, we are the first to report that the phenomenon and mechanism of joint IFN-γ and acidosis-inducible PD-L1 expression, representing a novel and targetable biomarker for identifying immune therapy responders.

### Supplementary Information

**Additional file 1: Fig. S1.** Induction of surrogate acidosis markers in MC38^wt^ cells treated with acidic cell culture media in vitro. **Fig. S2.** MC38^wt^ tumor cell proliferation after 72h incubation in acidic and neutral media. **Fig. S3.** A^IFN-γ^ induces PD-L1 expression on cancer cells. **Fig. S4.** IFN-γ induces *Stat1* and PD-L1 expression in CT26^wt^ cells. **Fig. S5.** Basal and IFN-γ-induced PD-L1 expression in murine wild-type cell lines and CRISPR/Cas9-generated PD-L1 knockout cells. **Fig. S6.** A^IFN-γ^ induces total and cell surface PD-L1 expression in different human cancer cell lines. **Fig. S7.** Imaging of tumor pH_e_ neutralization by NaHCO_3_ treatment using noninvasive in vivo acidoCEST MRI. **Fig. S8.** Histopathology of murine MC38^wt^, CT26^wt^, B16F10^wt^, 4T1^wt^ tumors. **Fig. S9.** CD3 Immunohistochemistry of murine CT26^wt^, B16F10^wt^ and 4T1^wt^ tumors. **Fig. S10.** Transcriptome correlation analysis of cutaneous melanoma samples of patients.

## Data Availability

The datasets analyzed during the current study are available from the corresponding author on reasonable request.

## Abbreviations

acidoCEST MRI: Acido-chemical exchange saturation transfer magnetic resonance imaging
eIF4F: Eukaryotic initiation factor 4F
FPKM: Fragments Per Kilobase of exon per Million reads
GAPDH: Glyceraldehyde 3-phosphate dehydrogenase
*i.c**.*: *Intracutaneous*
IFN-γ: Interferon-gamma
IHC: Immunohistochemistry
JAK1/2: Janus kinase 1/2
MFI: Mean fluorescence intensity
PD-1: Programmed death-1
PD-L1: Programmed death ligand-1
*s.c.*: *Subcutaneous*
siRNA: Small interfering RNA
STAT1/2: Signal transducer and activator of transcription 1/2
MMP-2/-9: Matrix metalloproteinase-2
MRS: Magnetic resonance spectroscopy
