# Supplementary material for: Acidosis-mediated increase in IFN-γ-induced PD-L1 expression on cancer cells as an immune escape mechanism in solid tumors
Source: Mol Cancer. 2023 Dec 15;22:207. doi: 10.1186/s12943-023-01900-0 (PMC10722725; doi:10.1186/s12943-023-01900-0)
Supplement: Supplementary file 1 — Additional file 1: Fig. S1. Induction of surrogate acidosis markers in MC38wt cells treated with acidic cell culture media in vitro. Fig. S2. MC38wt tumor cell proliferation after 72h incubation in acidic and neutral media. Fig. S3. AIFN-γ induces PD-L1 expression on cancer cells. Fig. S4. IFN-γ induces Stat1 and PD-L1 expression in CT26wt cells. Fig. S5. Basal and IFN-γ-induced PD-L1 expression in murine wild-type cell lines and CRISPR/Cas9-generated PD-L1 knockout cells. Fig. S6. AIFN-γ induces total and cell surface PD-L1 expression in different human cancer cell lines. Fig. S7. Imaging of tumor pHe neutralization by NaHCO3 treatment using noninvasive in vivo acidoCEST MRI. Fig. S8. Histopathology of murine MC38wt, CT26wt, B16F10wt, 4T1wt tumors. Fig. S9. CD3 Immunohistochemistry of murine CT26wt, B16F10wt and 4T1wt tumors. Fig. S10. Transcriptome correlation analysis of cutaneous melanoma samples of patients. [file 12943_2023_1900_MOESM1_ESM.pdf]

Supplemental Data

Figure S1

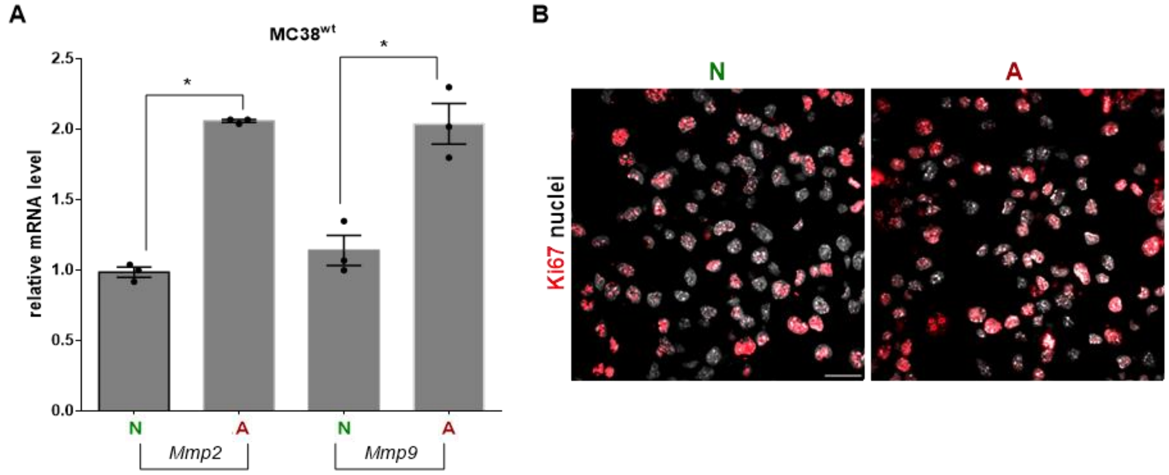

Fig. S1 Induction of surrogate acidosis markers in MC38^{wt} cells treated with acidic cell culture media *in vitro*. (A) Relative *Mmp2* and *Mmp9* mRNA expression normalized to *Gapdh*, *Aldolase* and *β-actin* (n = 3, statistics: two-tailed Student's t-test), both of which are surrogate markers for acidosis, and **(B)** fluorescent staining for Ki67 (red) and nuclei (gray) in MC38^{wt} cells treated with acidic cell culture media for 24 h. Scale bar: 20 µm. The data are presented as the means ± SEM. Abbreviations: N = neutral media, A = acidic media. Statistics: one-tailed Mann-Whitney test.

Figure S2

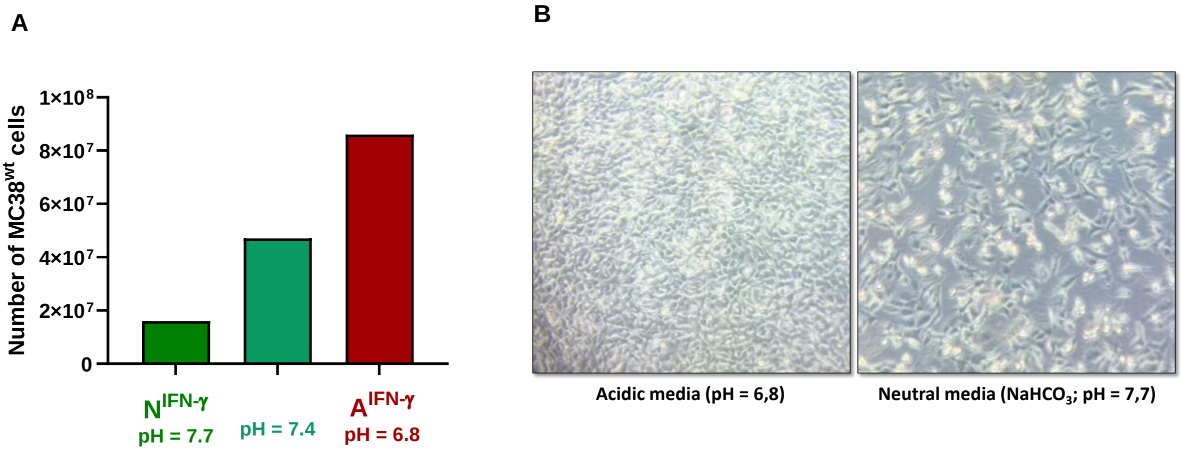

Fig. S2 MC38^{wt} tumor cell proliferation after 72h incubation in acidic and neutral media. (A) Number of MC38^{wt} tumor cells after 72h cultivation in acidic (pH = 6.8), intermediate (pH = 7.4) and neutral (pH = 7.7) media. Initially 5x10^{6} MC38^{wt} tumor cells were cultured as described in a T175 culture flask and counted after 72h. **(B)** Visualization of the MC38^{wt} cell density by light microscopy after 72h cultivation in acidic and neutral culture conditions (magnitude: 5x).

Figure S3

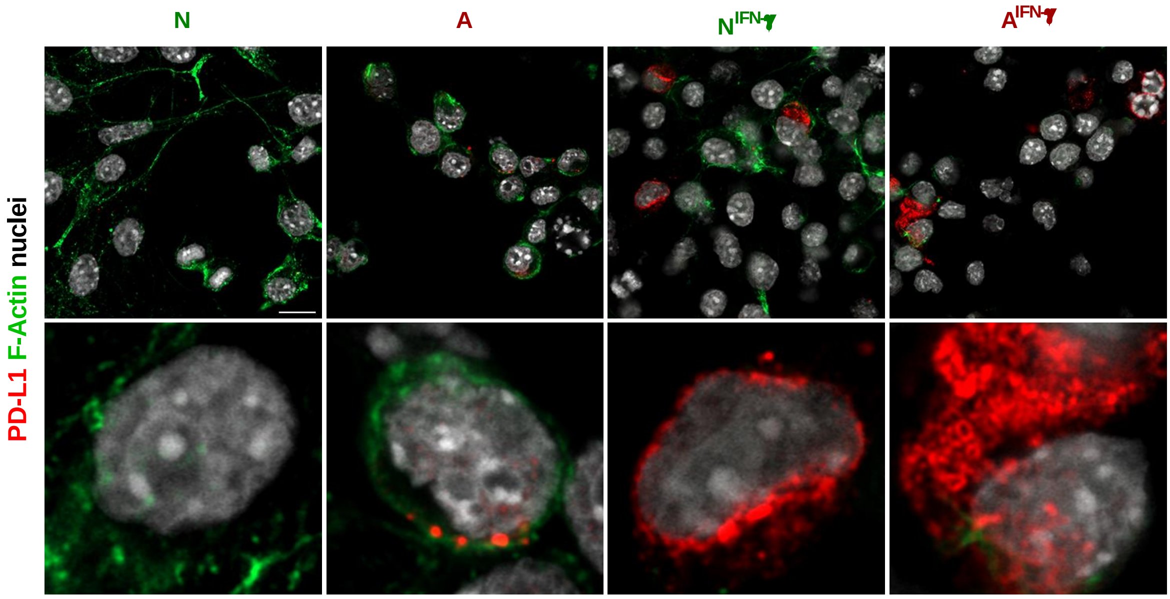

Fig. S3 A$^{IFN\text{-}\gamma}$ induces PD-L1 expression on cancer cells. Fluorescent staining for PD-L1 (red), F-actin (green) and nuclei (gray) in MC38^{wt} cells cultured with neutral or acidic cell culture media in the presence or absence of IFN-γ (10 ng ml^{-1}) for 72 h. Scale bar for the upper row: 10 µm. The lower row presents magnified images of a single cell from the images in the upper row.

Figure S4

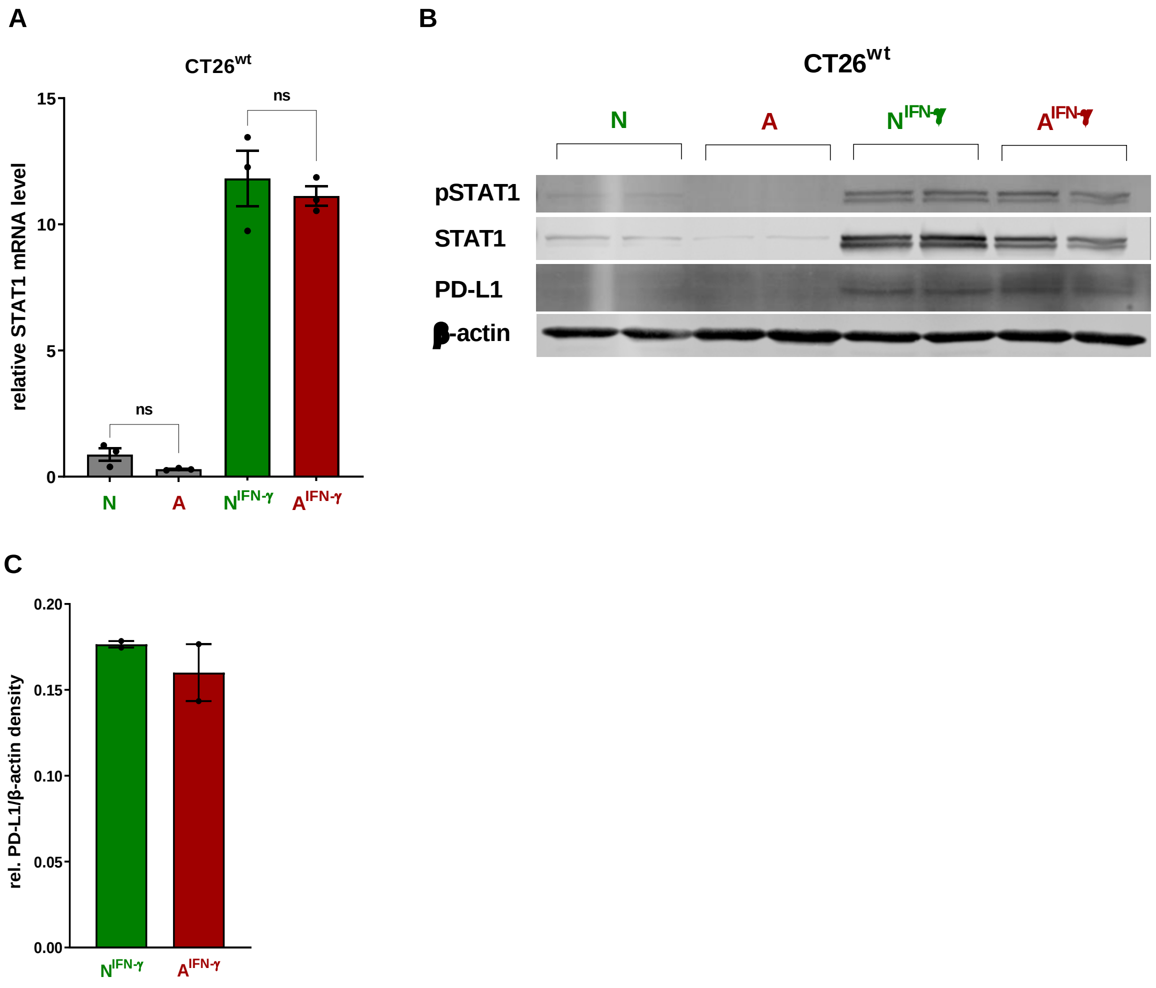

Fig. S4 IFN-γ induces *Stat1* and PD-L1 expression in CT26^{wt} cells. (A) Relative *Stat1* mRNA expression normalized to *Gapdh*, *Aldolase* and *β-actin* (n = 3, statistics: Tukey's multiple comparison test) and **(B)** Western blot analysis of PD-L1 and β-actin levels in CT26^{wt} cells treated with acidic media and/or IFN-γ (10 ng ml^{-1}) for 72 h (n = 2). Data are presented as the means ± SEM. N = neutral media, A = acidic media, N$^{IFN\text{-}\gamma}$ = neutral media plus IFN-γ, A$^{IFN\text{-}\gamma}$ = acidic media plus IFN-γ. **(C)** Densitometry of PD-L1 and β-actin levels from Western blotting of **B**.

Figure S5

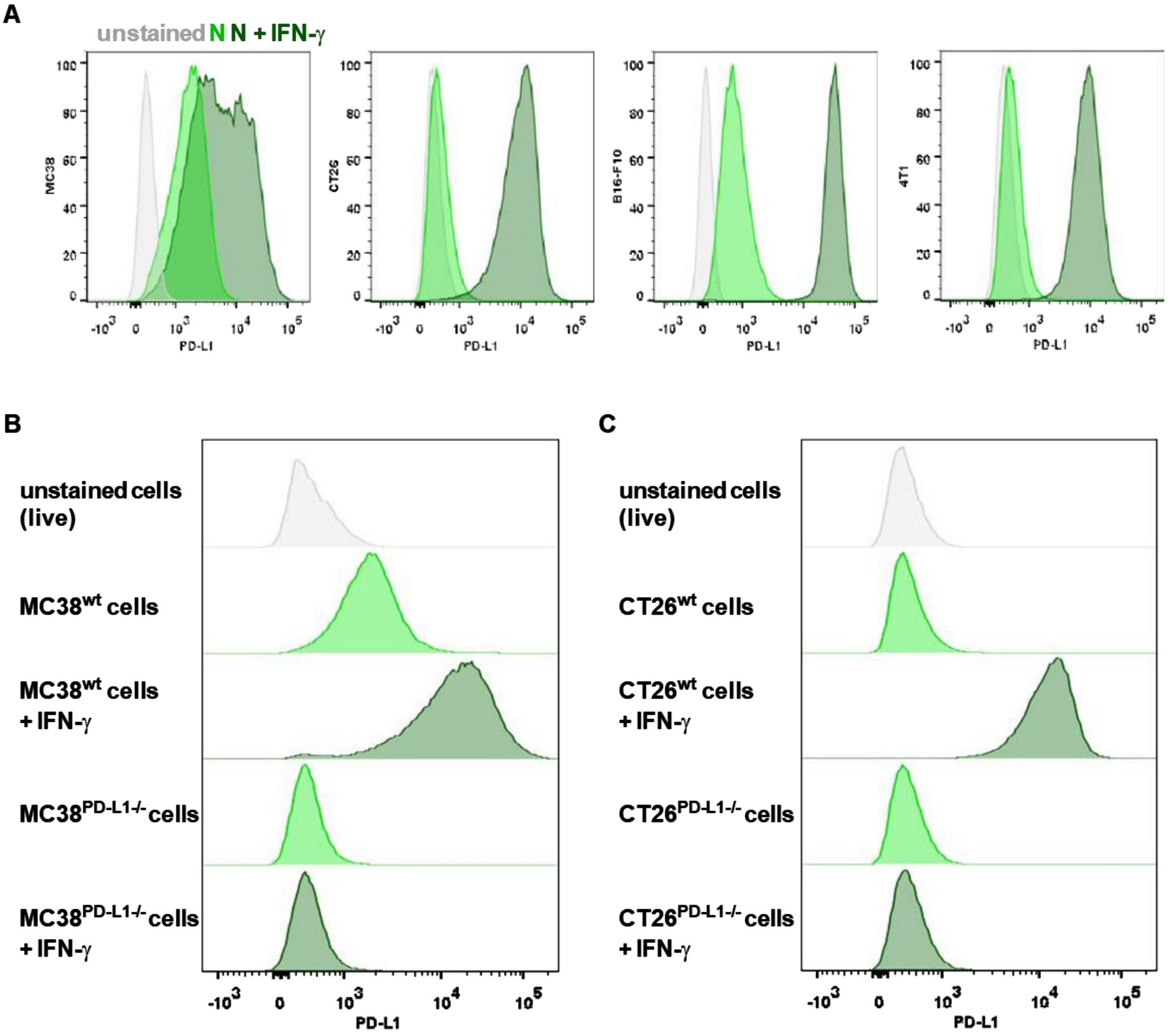

Fig. S5 Basal and IFN-γ-induced PD-L1 expression in murine wild-type cell lines and CRISPR/Cas9-generated PD-L1 knockout cells. (A) Basal (light green) and IFN-γ-induced (10 ng ml^{-1}; 24 h; dark green) PD-L1 expression in MC38^{wt}, CT26^{wt}, B16-F10^{wt} and 4T1^{wt} cells visualized using flow cytometry. Unstained live cells are shown in gray. Histograms of PD-L1 expression on **(B)** MC38^{wt} and MC38$^{PD\text{-}L1-/-}$ and **(C)** CT26^{wt} and CT26$^{PD\text{-}L1-/-}$ cells cultured in the absence (light green) or presence (dark green) of IFN-γ (100 ng ml^{-1}). Cells were stimulated for 24 h (MC38^{wt} and MC38$^{PD\text{-}L1-/-}$) or 48 h (CT26^{wt} and CT26$^{PD\text{-}L1-/-}$) with IFN-γ; unstained living cells (gray) served as controls.

Figure S6

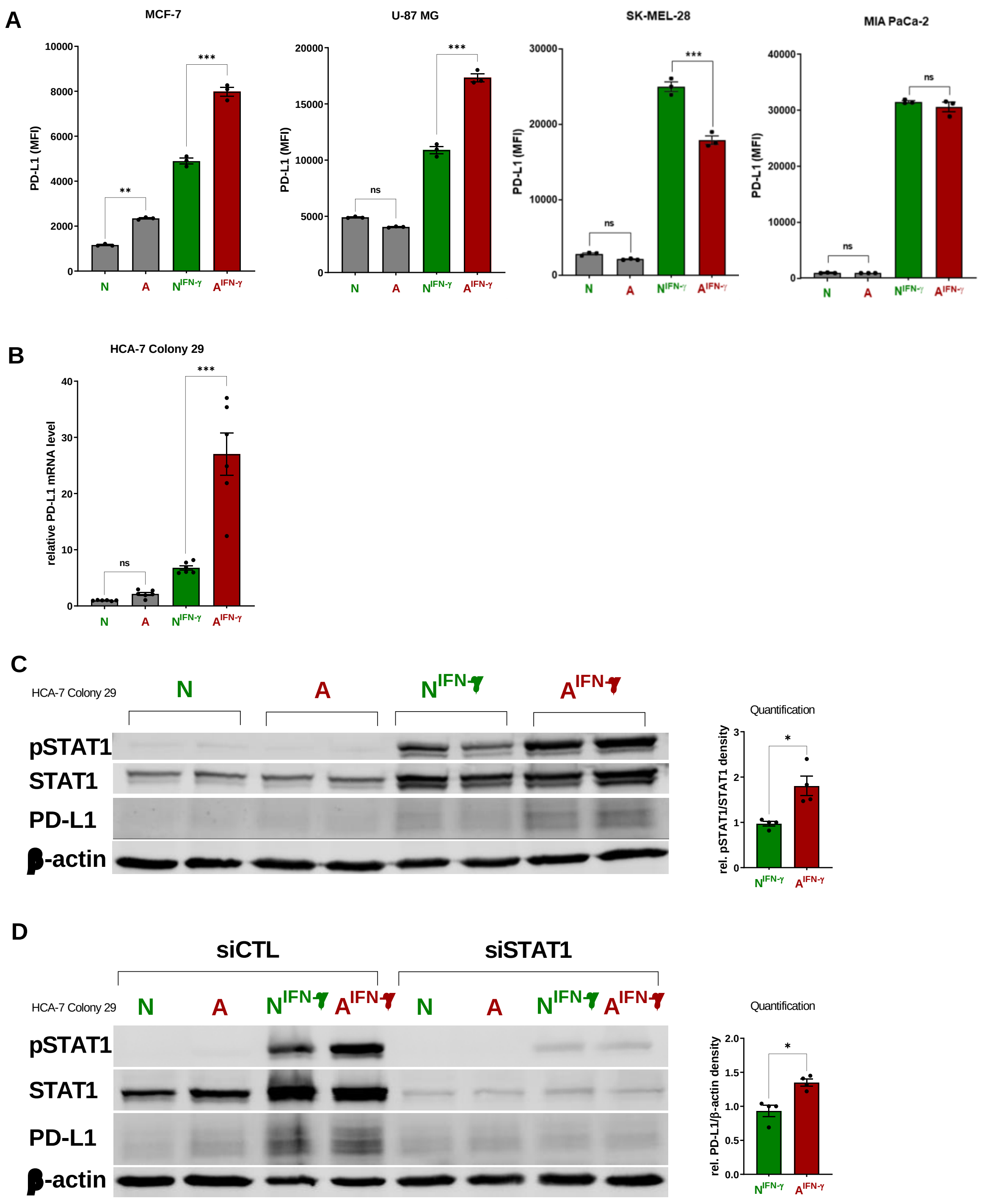

Fig. S6 A$^{IFN\text{-}\gamma}$ induces total and cell surface PD-L1 expression in different human cancer cell lines. (A) PD-L1 MFI measured using flow cytometry in human MCF-7, U-87 MG, SK-

MEL-28 and MIA PaCa-2 cells treated with acidic media and/or IFN-γ (10 ng ml^{-1}) for 72 h (n = 3, statistics: Tukey's multiple comparison test). **(B)** Relative *Stat1* and *CD274* mRNA expression normalized to *Gapdh*, *Aldolase* and *β-actin* (pooled data from 2 experiments, n = 5-6, statistics: Tukey's multiple comparison test) and **(C-D)** Western blot analyses and densitometry of pSTAT1, STAT1, PD-L1 and β-actin levels in the HCA-7 colony 29 cell line treated with acidic media and/or IFN-γ (10 ng ml^{-1}) for 72 h (pooled data from 2 experiments, n = 4, statistics: nonparametric Mann-Whitney test). HCA-7 colony 29 cells were transfected with a control (siCTL) or STAT1-specific siRNA and treated with acidic media and/or IFN-γ (10 ng ml^{-1}) for 24 h. **(E)** Western blot analyses of pSTAT1, STAT1, PD-L1 and β-actin levels were performed to determine the STAT1 knockdown efficiency and absence of PD-L1 induction following treatment with the combination of IFN-γ and acidic media (n = 2). Data are presented as the means ± SEM. N = neutral media, A = acidic media, $N^{IFN\text{-}\gamma}$ = neutral media plus IFN-γ, $A^{IFN\text{-}\gamma}$ = acidic media plus IFN-γ.

Figure S7

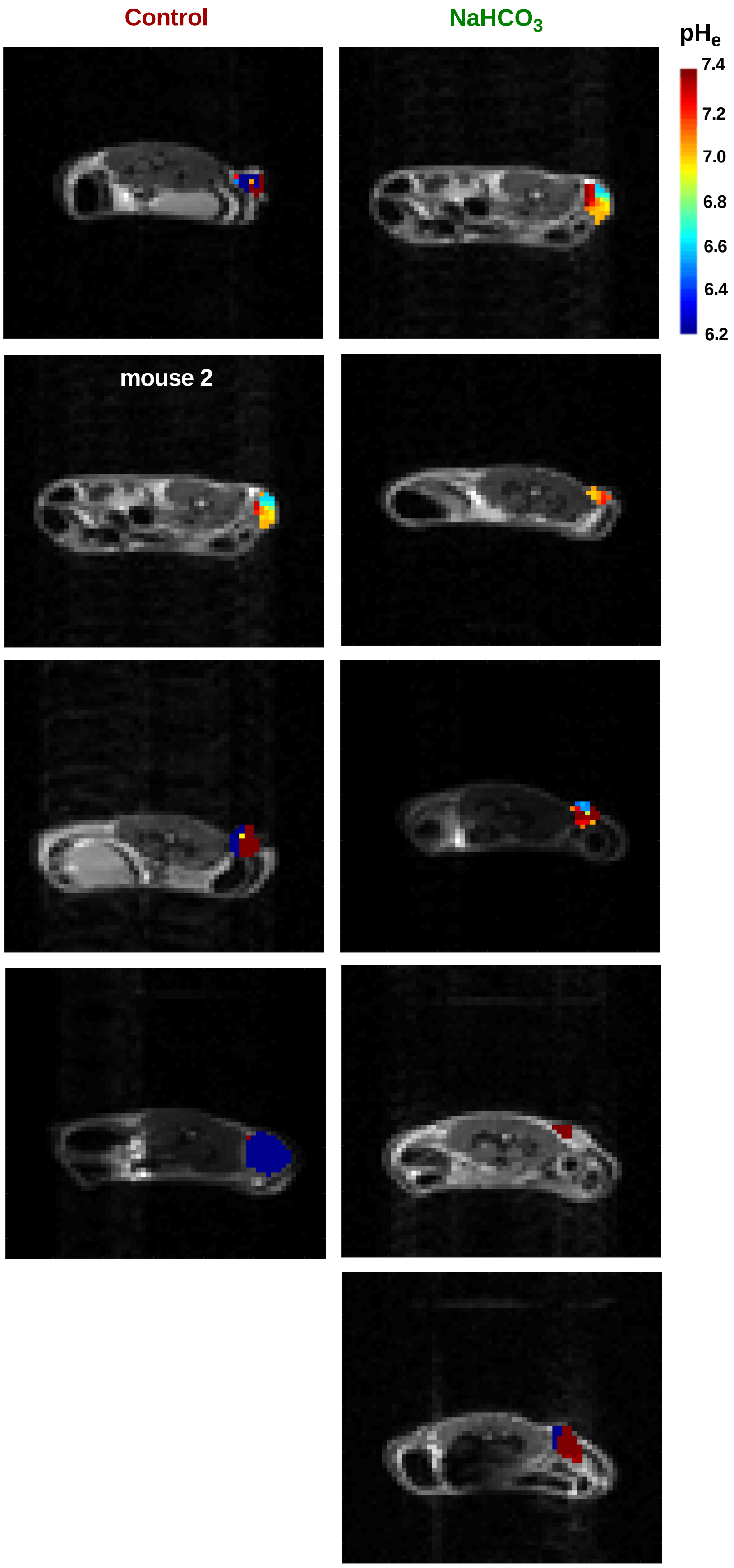

Fig. S7 Imaging of tumor pH_e neutralization by $NaHCO_3$ treatment using noninvasive *in vivo* acidoCEST MRI. $NaHCO_3$-enriched drinking water increased the tumor pH_e in mice ($NaHCO_3$) compared to mice receiving regular drinking water, where the tumor pH_e is acidic (control). Representative pH_e maps overlaid on T_2-weighted axial MRI images of $MC38^{wt}$ tumor-bearing mice injected with iopamidol (*i.v.*) at day 10 after the cancer cell injection. Mice received either $NaHCO_3$ (200 mM) water or regular drinking water (control) three days prior to cancer cell inoculation. Mouse 4 from the $NaHCO_3$ group and mouse 4 from the control group were excluded from the analysis, as the measured pH_e value was out of the calibration range.

Figure S8

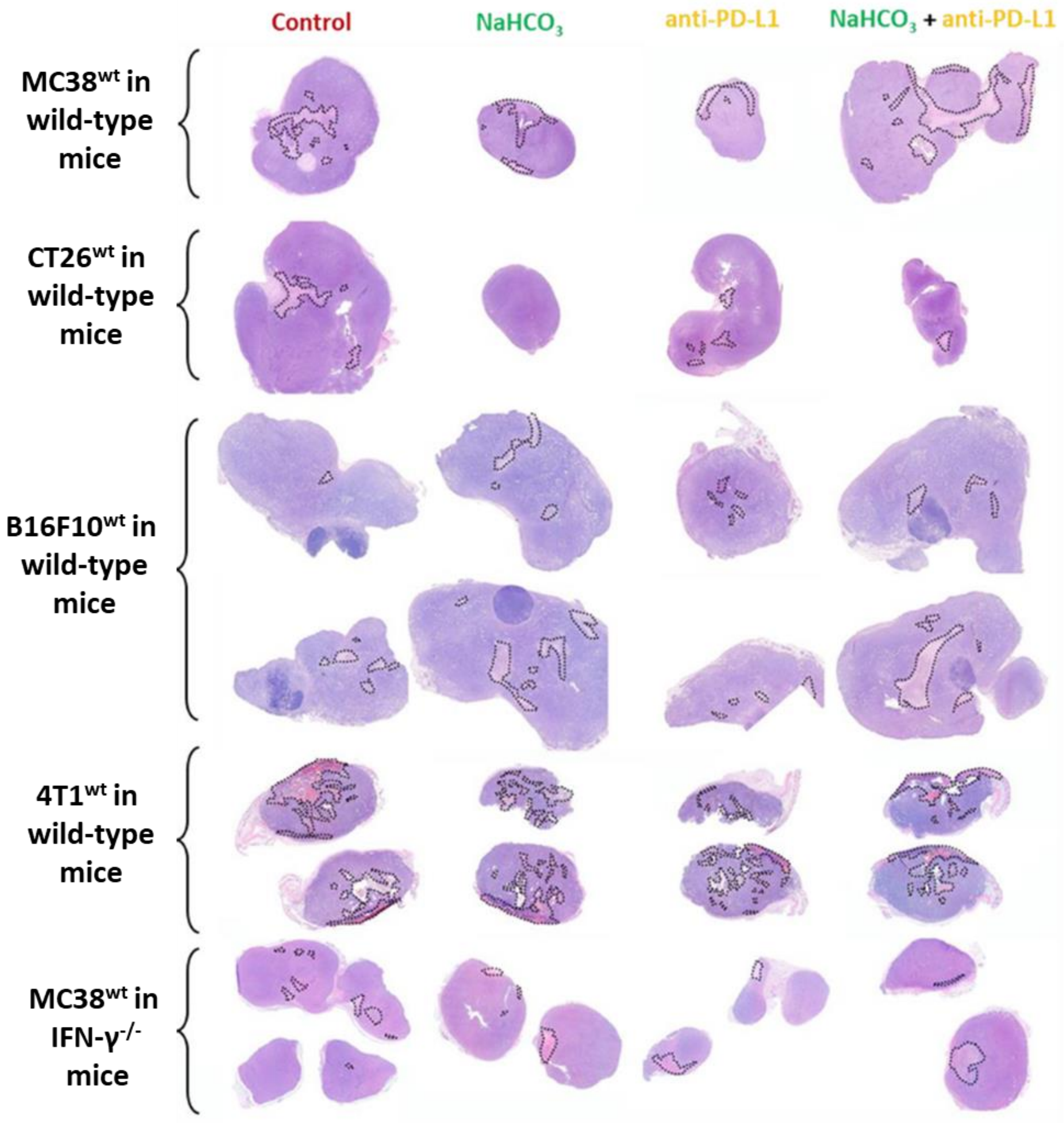

Fig. S8 Histopathology of murine MC38^{wt}, CT26^{wt}, B16F10^{wt}, 4T1^{wt} tumors. Representative H&E staining of *s.c.* MC38^{wt}, CT26^{wt}, B16F10^{wt} and 4T1^{wt} tumors derived from C57BL/6J wild-type mice and of MC38^{wt} tumors derived from IFN-$\gamma^{-/-}$ mice after the different treatment conditions. Necrotic areas are encircled by a dashed black line. Animals received regular drinking water (control), $NaHCO_3$ in water ($NaHCO_3$), anti-PD-L1 mAb or $NaHCO_3$ & anti-PD-L1 mAb. Tumors from n = 7 – 8 animals per treatment group underwent histopathological analysis by a professional pathologist.

Figure S9

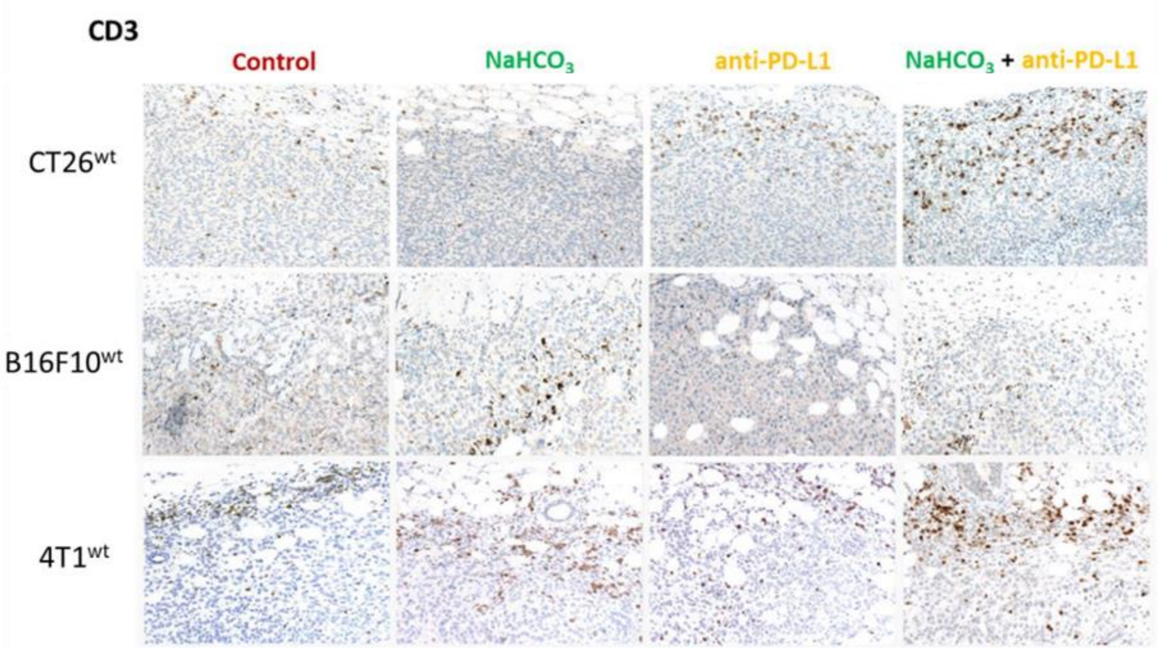

Figure S9 CD3 Immunohistochemistry of murine CT26^{wt}, B16F10^{wt} and 4T1^{wt} tumors. Representative CD3 immunohistochemistry of *s.c.* CT26^{wt}, B16F10^{wt} and 4T1^{wt} and tumors derived from wild-type C57BL/6J mice at day 17 (CT26^{wt}), day 14 (B16F10^{wt}) and day 20 (4T1^{wt}) after different treatment conditions. Animals received regular drinking water (control), $NaHCO_3$ in water ($NaHCO_3$), anti-PD-L1 mAb or $NaHCO_3$ & anti-PD-L1 mAb. N = 3 – 4 representative tumors of each experimental group were subjected to CD3 staining which was analyzed by a professional pathologist.

Figure S10

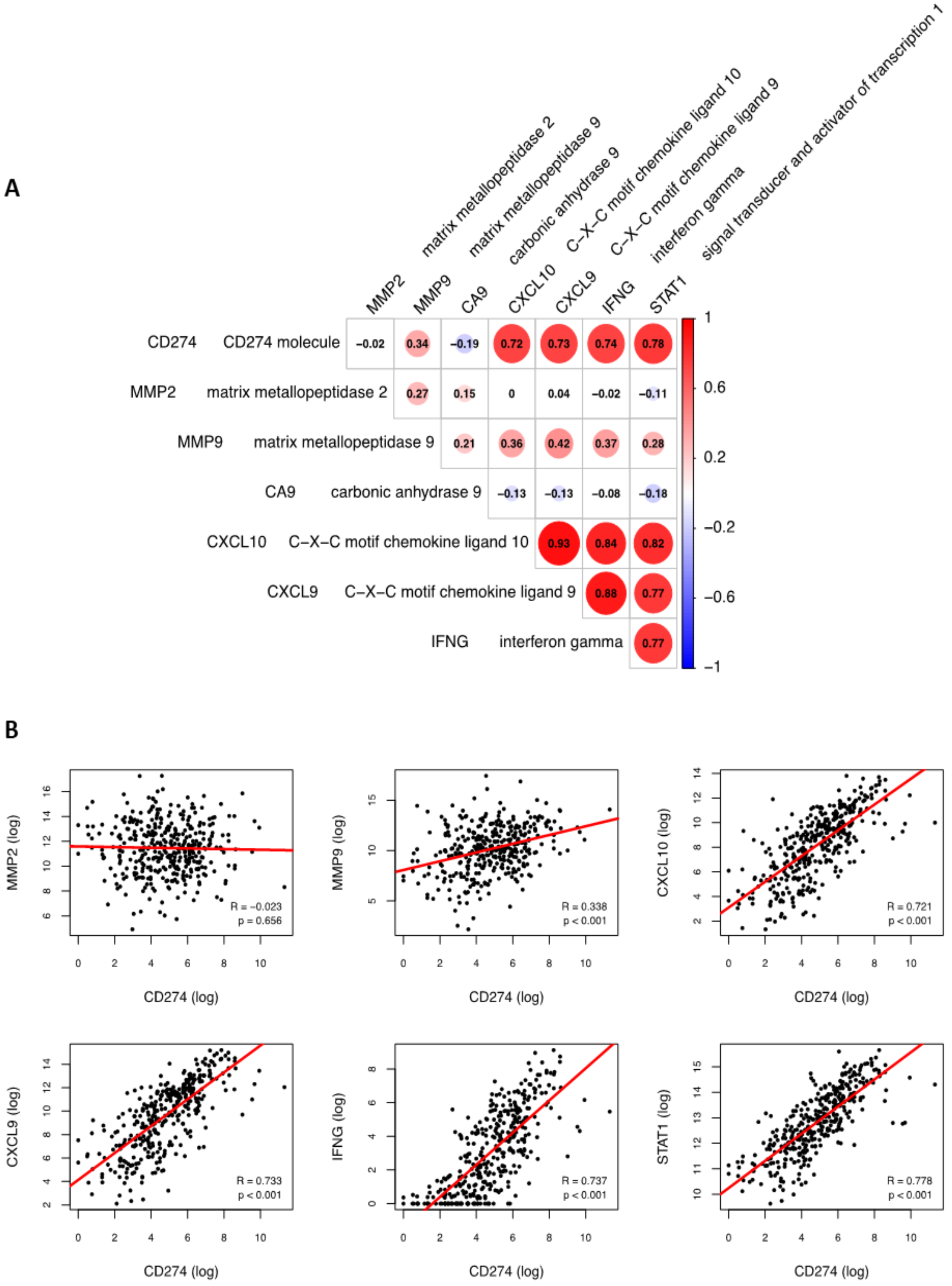

Figure S10 Transcriptome correlation analysis of cutaneous melanoma samples of patients. (A) The Cancer Genome Atlas (TCGA) RNA-seq data were analyzed to determine correlations between IFN-γ, STAT1, CXCL10, CXCL9, MMP2, MMP9, CA9, and CD274

(PD-L1) gene expression in cutaneous melanoma samples from 368 patients. Numbers represent the Pearson coefficients, statistically significant results ($p < 0.05$) are indicated by the presence of a colored circle. Scatter plots represent expression data across all 368 samples. The results are based on data generated by TCGA Research Network: https://www.cancer.gov/tcga. (B) Correlation plots of CD274 (PD-L1) to the genes IFN-γ, STAT1, CXCL10, CXCL9, MMP2 and MMP9.

Supplementary References

1. Mall, C., et al., *Repeated PD-1/PD-L1 monoclonal antibody administration induces fatal xenogeneic hypersensitivity reactions in a murine model of breast cancer.* Oncoimmunology, 2016. **5**(2): p. e1075114.
